# Impact pathways of a homestead food production programme on women’s dietary diversity in Bangladesh

**DOI:** 10.1038/s43016-026-01354-9

**Published:** 2026-05-12

**Authors:** Nathalie J. Lambrecht, Thalia M. Sparling, Axel Mayer, Jillian L. Waid, Amanda S. Wendt, Masum Ali, Sabine Gabrysch

**Affiliations:** 1 Research Department 2, Potsdam Institute for Climate Impact Research, Member of the Leibniz Association, Potsdam, Germany; 2 Institute of Public Health, Charité – Universitätsmedizin Berlin, Corporate Member of Freie Universität Berlin and Humboldt-Universität zu Berlin, Berlin, Germany; 3 Center for Innovation in Global Health, Stanford University, Stanford, CA USA; 4 London School of Hygiene and Tropical Medicine, London, UK; 5 Bielefeld University, Bielefeld, Germany; 6 Nutrition and Health Sciences, Laney Graduate School, Emory University, Atlanta, GA USA; 7 Heidelberg Institute of Global Health, Medical Faculty and University Hospital, Heidelberg University, Heidelberg, Germany

**Keywords:** Malnutrition, Agriculture, Developing world

## Abstract

Homestead food production (HFP) can improve nutrition through multiple pathways. Understanding their relative importance can optimize intervention design and impact. Here we used panel data on 2,612 women from a 1:1 cluster-randomized trial of 96 settlements in rural Bangladesh and conducted multiple mediation analysis to investigate the impact pathways of a 3-year HFP intervention on women’s dietary diversity. The pathways analysed fully explained the intervention’s total effect on dietary diversity score, amounting to an increase of 0.4 (95% confidence interval (CI) 0.3–0.5) food groups on a 10-point scale. Garden production accounted for 78% of the increase (*β* = 0.33 (95% CI 0.25–0.42)), emerging as the key component to improving dietary diversity. Nutrition knowledge accounted for 18% of the increase, while poultry production and market activity made smaller contributions. These findings can guide the design of future HFP interventions, but similar analyses are needed across a range of interventions, outcomes and settings to build a robust evidence base for improving nutrition.

## Abstract (web summary)

The contribution of homestead food production (HFP) to nutrition and dietary diversity is context-specific and involves multiple pathways. Using panel data on 2,612 women in 96 settlements in rural Bangladesh, this study identifies and measures the impact pathways of a 3-year HFP intervention.

## Main

Approximately 3.1 billion people (42% of the world’s population) cannot afford adequate, healthy diets^1^. In South Asia, which includes some of the world’s most densely populated countries, this figure is almost 1.4 billion—three-quarters of the regional population^1^. Consumption of staple-heavy diets contributes to micronutrient deficiencies and short- and long-term health risks, including impaired growth and development, anaemia, susceptibility to infectious diseases, and non-communicable diseases^2–4^.

Nutrition-sensitive agricultural interventions aim to address the underlying determinants of poor nutrition by integrating nutrition goals into agricultural production^5^. Homestead food production (HFP) programmes are the most common nutrition-sensitive agriculture interventions and, at their core, promote home gardening and/or small livestock rearing to increase small-scale food production and diversified food consumption^6^. In theory, improvements in diets can stem from increased consumption of own farm production, or from increased income and purchasing power at markets when crops and livestock products are sold. Many studies have found evidence for the link between higher production diversity and dietary diversity, with stronger associations observed in settings characterized by limited market access^7,8^.

Reviews published on nutrition-sensitive agricultural interventions find that HFP programmes generally have positive impacts on production diversity and some impacts on dietary intake; however, there is limited evidence of impacts on downstream health outcomes, such as child growth and development, partly due to inadequate study designs, sample sizes and study durations^5,9–12^. HFP interventions are inherently complex, typically incorporating multiple programme components beyond food production to achieve nutrition and health impacts (for example, nutrition and health education; water, sanitation and hygiene; gender equity; and marketing activities)^6^. While the complexity of these interventions holds potential for greater impacts, it also makes it difficult to disentangle which components are most effective at impacting intended outcomes.

The use of programme impact pathway (PIP) diagrams early in the research process can describe hypothesized causal pathways to impact, facilitate monitoring and evaluation along these pathways, and help trace how interventions contribute to intended outcomes^13,14^. Several recent large-scale multicomponent HFP studies have incorporated PIPs in their trial design^15–19^. However, quantitative estimation of impact pathways is rare so far, and has primarily been limited to evaluating effects on intermediate outcomes (for example, agricultural production) along the causal pathway^17,18,20–23^ or single mediator analyses^24–26^. As such, it remains unclear whether HFP interventions improve diets primarily through increases in production diversity, marketing, knowledge or women’s empowerment, for instance, due to a lack of analyses that simultaneously examine multiple pathways.

Structural equation modelling (SEM) is a valuable tool for estimating PIPs as it allows researchers to model impacts using multiple mediators. Recent methodological developments have strengthened our understanding of the assumptions underlying causal inference from these models—most importantly, linear relationships, the absence of exposure–mediator interactions, and no unmeasured confounding—as well as their connections to the definitions of natural direct and indirect effects from the causal inference literature^27–30^. SEM in conjunction with high-quality and frequent data on intermediate outcomes can enable researchers to more effectively evaluate PIPs of complex interventions and inform improved intervention design and delivery. To our knowledge, only three studies have used multiple mediation analysis to examine the impact pathways of HFP interventions, all based on data collected only at baseline and endline or post-endline^31–33^.

We fill this gap by conducting a mediation analysis of the multiple impact pathways of a 3-year HFP intervention in Bangladesh on women’s dietary diversity, leveraging a large sample size, a randomized design and frequent high-quality data collection on intermediate outcomes. The HFP intervention was evaluated as part of the Food and Agricultural Approaches to Reducing Malnutrition (FAARM) cluster-randomized trial and aimed to improve diets and reduce undernutrition in women and their children. The intervention group received training on the production of garden crops and poultry products, plus food hygiene and nutrition behaviour change communication. At the start of the third year of the intervention, marketing training was provided for women who were able to produce surplus garden and poultry products. Several outcomes of the FAARM trial have been previously published: Women’s and children’s dietary diversity increased by an average of 0.4 (out of 10) and 0.3 food groups (out of 7), respectively, with increases in nearly all food groups, including dark-green leafy vegetables, other vitamin-A-rich vegetables, other fruits, legumes, nuts and seeds, and eggs^34^. There was a substantial increase in harvested garden crop species, especially among the 22 promoted vegetable species, including green amaranth, Indian spinach, yardlong bean, red pumpkin and various gourds^35^, in poultry and egg production^24^, as well as women’s intrinsic and collective agency^36^.

**Fig. 1:**
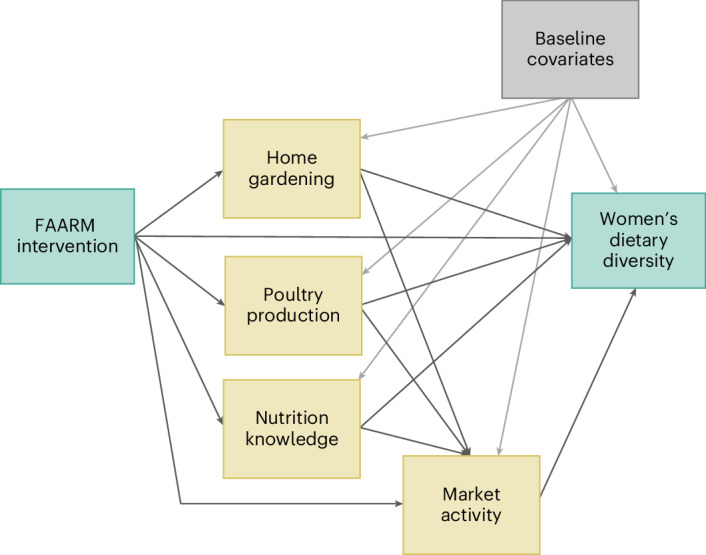
DAG for the impact pathways of the FAARM intervention. The green boxes represent the exposure and the outcome, the yellow boxes represent the mediator domains (home gardening, poultry production, women’s nutrition knowledge and women’s market activity) and the grey box represents potential confounders. We specified direct paths from the intervention to the outcome and mediators. Women’s market activity was also regressed on the garden, poultry and knowledge mediators to include the indirect paths through this domain. Inclusion of baseline covariates controlled for hypothesized mediator–outcome confounding.

In this study, we quantified the key impact pathways contributing to improved dietary diversity among women in a multiple mediation model. We used high-frequency panel data from the FAARM surveillance system’s routine assessment of dietary diversity, garden production and poultry production as well as trial endline data to evaluate effects in the latter half of the trial, when intervention activities should have reached their fullest extent. We conducted causal mediation analyses using SEM to assess the direct and indirect effects of the intervention on women’s average dietary diversity score (DDS) and the proportion of times women met minimum dietary diversity (MDD) based on a directed acyclic graph (DAG; Fig. 1). Mediators to capture the key domains of the intervention comprised indicators of home gardening (crop species richness and garden practices score), poultry production (number of poultry owned and poultry eggs produced) and women’s nutrition knowledge (food group knowledge score and diet diversity knowledge score). We also included pathways through women’s market activity (whether women bought or sold goods, and whether women went to the market to buy goods). In an additional analysis, we explored pathways through women’s empowerment, given women’s limited mobility and agency in the study setting. Moreover, we assessed effect heterogeneity across household religion, household wealth and women’s education. Further details on variable and model specifications are provided in the Methods.

## Results

We enrolled 2,705 women in the FAARM trial (control 1,368, intervention 1,337; Supplementary Section 1) and reached 2,620 women during surveillance and 2,578 at endline, 1,291 (94%) in the control and 1,287 (96%) in the intervention arm. Our total analytic sample size consisted of 2,612 women (control 1,312, intervention 1,300). Women were excluded if they did not provide any outcome data during the analysed time period (*n* = 93).

Women were, on average, 25 years old at baseline, and just under two-thirds had completed at least a primary school education (Table 1). Two-thirds were Muslim and one-third Hindu. At baseline, women consumed, on average, four food groups in a day, and 31% consumed a minimally diverse diet, that is, at least five out of ten food groups. Women scored on the low end in several domains of empowerment. Mobility was particularly low, with less than a third of women having left their homestead at least once in the month before the interview. Baseline characteristics were similar between the trial arms (Table 1).

**Table 1:** Baseline characteristics of women participating in the FAARM trial in Bangladesh

Characteristic	Control		Intervention	
	*n*	% or µ ± s.d.	*n*	% or µ ± s.d.
Woman’s DDS (0–10 food groups)	1,257	3.9 ± 1.4	1,254	3.9 ± 1.3
Woman met MDD (5+ food groups)	1,257	30.7	1,254	31.1
Household religion	1,312		1,300	
Muslim		66.1		70.5
Hindu		33.9		29.5
Household wealth quintile	1,308		1,293	
Lowest		25.9		21.8
Low		23.7		19.8
Middle		19.3		20.1
High		15.9		20.3
Highest		15.2		17.9
Homestead land size, m^2^	1,308	546 ± 700	1,292	666 ± 921
Agricultural land size, m^2^	1,308	5,969 ± 16,812	1,292	6,800 ± 15,014
Garden crop species richness	1,308	5.8 ± 4.4	1,292	6.6 ± 4.7
Woman’s age, years	1,257	24.4 ± 4.4	1,254	24.6 ± 4.3
Woman’s education level	1,312		1,300	
None		15.8		15.1
Partial primary		22.6		21.4
Complete primary		23.8		21.9
Partial secondary		32.8		35.0
Complete secondary		5.0		6.6
Market activity score [0–3]	1,305	0.5 ± 0.7	1,291	0.5 ± 0.7
Woman’s empowerment				
Social support score [0–2]	1,302	1.7 ± 0.4	1,289	1.7 ± 0.4
Communication with husband score [0–2]	1,296	1.8 ± 0.4	1,282	1.8 ± 0.4
External communication score [0–2]	1,302	0.2 ± 0.4	1,289	0.2 ± 0.4
Decision-making score [0–2]	1,302	0.6 ± 0.6	1,289	0.7 ± 0.6
Mobility: left homestead in prior month	1,302	31.8	1,289	29.9
Own income: earned money in prior month	1,302	10.7	1,289	10.5

Characteristics are presented by intervention arm for the analytic sample. Values are percentages for categorical variables or µ (mean) ± s.d. for continuous variables. Scores ranges are given in brackets.

The FAARM intervention had a positive impact on women’s DDS, MDD and all of the pathway indicators (that is, mediators) over the analysed time period (Table 2), consistent with impacts reported earlier^24,34,35^. In the latter half of the trial, DDS had increased by 0.5 food groups (out of 10) over control and an additional 13% of women met MDD. There was strong evidence for improvements in garden production, with women in the intervention arm harvesting, on average, 5.4 more crop species and engaging in 4.8 more promoted garden practices compared with the control arm. Women also reared 1 additional poultry bird and produced 1.7 more eggs in the past week. The intervention also increased women’s nutrition knowledge, both on diet diversity (0.4 points more out of 4) and on the health benefits of selected food groups (0.6 more correct answers out of 5). By the end of the trial, the intervention increased women’s market activity score by 0.3 points on a 3-point scale.

**Table 2:** Intervention impacts on women’s dietary diversity and the mediators

Indicator	*n*	Con.	Int.	Effect estimate	95% CI	*P* value
DDS	2,612	4.5	5.0	0.5	0.3 to 0.6	<0.001
Met MDD	2,612	0.46	0.58	0.13	0.08 to 0.18	<0.001
Crop species richness	2,465	5.4	10.7	5.4	4.5 to 6.3	<0.001
Garden practices score	2,389	4.6	9.3	4.8	4.0 to 5.5	<0.001
Number of poultry owned	2,447	2.2	3.3	1.0	0.4 to 1.6	0.001
Number of eggs produced in past week	2,447	3.0	4.7	1.7	0.7 to 2.7	0.001
Food group knowledge score	1,765	1.8	2.4	0.6	0.4 to 0.7	<0.001
Diet diversity knowledge score	2,363	2.6	3.0	0.4	0.3 to 0.6	<0.001
Market score	2,337	0.6	0.9	0.3	0.1 to 0.4	<0.001

Values for control and intervention group are descriptive means. Effect estimates (*β*) and two-sided *P* values are calculated using mixed-effects linear regression, with random effects to control for clustering at the settlement level. The models for DDS and met MDD control for baseline DDS, the model for crop species richness controls for baseline crop species richness, and the model for market score controls for baseline market score. Each model included all 96 settlements. Variable values were averaged over survey rounds in the second half of the trial, with different numbers of rounds included based on data availability. Descriptions of each variable and the time period analysed are provided in Methods and Supplementary Section 15. All variables are at the woman level, that is, women’s diets, gardening, poultry, nutrition knowledge and market activity. DDS measures the number of food groups consumed in the previous 24 h and ranges from 0 to 10, and MDD is defined as consuming at least 5 out of 10 food groups. The garden practices score included 17 practices, the food group knowledge score ranges from 0 to 5 points, the diet diversity knowledge score ranges from 0 to 4 points, and the market score ranges from 0 to 3 points. Con., control; Int., intervention.

**Fig. 2:**
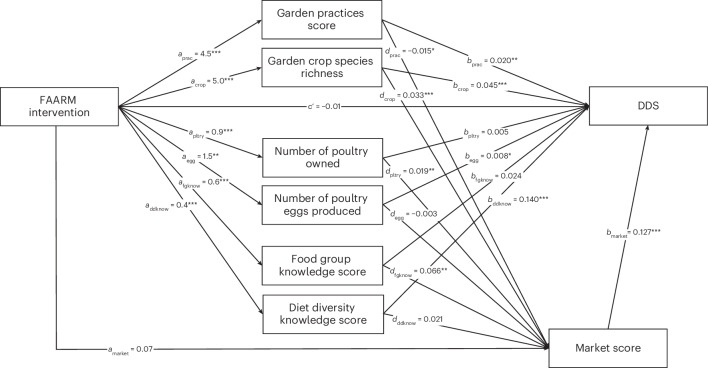
Causal mediation pathways of the FAARM intervention on women’s DDS. Path coefficients estimated using structural equation models (*n* = 2,612) with full information maximum likelihood and cluster-adjusted standard errors, controlling for baseline covariates. Unstandardized coefficients of the *a* paths are the effect of the intervention on the mediators, coefficients of the *b* paths are the effect of a one-unit increase in the mediators on DDS, and coefficients of the *d* paths are the effect of a one-unit increase in the mediators on women’s market score. Residual correlations and variances are omitted from the figure. Corresponding indirect effects and 95% confidence intervals are provided in Table 3, and exact *P* values are reported in Supplementary Section 3. *P* values are estimated using two-sided Wald tests. *P < 0.05, **P < 0.01, ***P < 0.001.

The results from separate regression models are congruent with the *a* paths in the mediation model that quantifies the simultaneous effects of the intervention on each domain (Fig. 2). The *b* paths of the mediation model, from mediators to dietary diversity (Fig. 2), show that garden crop species richness, garden practices, diet diversity knowledge and market score had strong positive effects on DDS. For each additional crop species harvested, women’s dietary diversity increased by 0.05 food groups. Poultry egg production also had a small positive effect on DDS. Standardized estimates of the *a* and *b* paths are provided in Supplementary Section 2.

The models for the primary outcomes demonstrated excellent fit based on the comparative fit index (CFI) and root mean square error of approximation (RMSEA), but suboptimal fit based on the Tucker–Lewis index (TLI) and the chi-square test (Table 3 and Supplementary Section 6), which probably reflects the complexity of the models as well as the chi-square test’s sensitivity to large sample sizes.

**Table 3:** Direct and indirect effects of the FAARM intervention on women’s DDS

	*β*	95% CI	Proportion mediated
Total effect^a^	0.423	0.238 to 0.602	
Direct effect	−0.010	−0.172 to 0.151	
Indirect effect^b^	0.433	0.338 to 0.533	102.4%
**Individual indirect effects:**			
**Home garden production**			
Garden practices score (*a*_prac_ × *b*_prac_)	0.092	0.024 to 0.166	
Garden crop species richness (*a*_crop_ × *b*_crop_)	0.226	0.159 to 0.301	
Garden practices via market (*a*_prac_ × *d*_prac_ × *b*_market_)	−0.009	−0.019 to −0.001	
Garden crops via market (*a*_crop_ × *d*_crop_ × *b*_market_)	0.021	0.010 to 0.035	
**Poultry production**			
Number of poultry owned (*a*_pltry_ × *b*_pltry_)	0.004	−0.009 to 0.018	
Number of poultry eggs produced (*a*_egg_ × *b*_egg_)	0.012	0.002 to 0.026	
Poultry owned via market (*a*_pltry_ × *d*_pltry_ × *b*_market_)	0.002	0.000 to 0.005	
Poultry eggs via market (*a*_egg_ × *d*_egg_ × *b*_market_)	−0.001	−0.003 to 0.001	
**Women’s nutrition knowledge**			
Food group knowledge score (*a*_fgknow_ × *b*_fgknow_)	0.015	−0.015 to 0.042	
Diet diversity knowledge score (*a*_ddknow_ × *b*_ddknow_)	0.056	0.026 to 0.092	
Food group score via market (*a*_fgknow_ × *d*_fgknow_ × *b*_market_)	0.005	0.001 to 0.011	
Diet diversity score via market (*a*_ddknow_ × *d*_ddknow_ × *b*_market_)	0.001	−0.001 to 0.004	
**Women’s market activity**			
Market score (*a*_market_ × *b*_market_)	0.009	−0.006 to 0.026	
Production/knowledge via market (∑*a* × *d* × *b* paths)	0.020	0.009 to 0.034	
**Indirect effects by domain:**			
Home garden production (∑prac + crop)	0.318	0.237 to 0.407	75.2%
Home garden production with market (∑prac + crop + garden prod via market)	0.329	0.247 to 0.422	78.0%
Poultry production (∑pltry + egg)	0.016	0.003 to 0.032	3.8%
Poultry production with market (∑pltry + egg + poultry prod via market)	0.018	0.004 to 0.033	4.2%
Nutrition knowledge (∑fgknow + ddknow)	0.070	0.028 to 0.116	16.7%
Nutrition knowledge with market (∑fgknow + ddknow + know via market)	0.077	0.034 to 0.123	18.1%
Market activity (∑market + prod/know via market)	0.029	0.012 to 0.051	6.8%

Estimates are unstandardized effects with 95% Monte Carlo confidence intervals. The structural equation model controlled for the following baseline covariates as potential mediator–outcome confounders: religion, household wealth quintile, log-transformed homestead land size, log-transformed agricultural land size, garden crop species richness, women’s market score, women’s education, domains of women’s empowerment (including mobility, social support, communication with husband, external communication, own income and decision-making) and women’s DDS. We estimated the model using full information maximum likelihood and allowed for correlations between the residuals of all the poultry and garden mediators and between the residuals of the knowledge mediators. The model includes 2,612 women. Fit statistics: CFI 0.993; TLI 0.834; RMSEA 0.045; *χ*^2^(8) = 49.8, *P* < 0.001. ^a^The total effect is the sum of the direct effect and the total indirect effect. ^b^The indirect effect is calculated as the sum of the indirect effects through garden production, poultry production, nutrition knowledge and market activity.

The impact pathways through garden production, poultry production, nutrition knowledge and market activity explained all of the FAARM intervention’s total effect on DDS (Table 3). The combined indirect effect of the intervention on DDS through these pathways was 0.43 food groups (95% confidence interval 0.34 to 0.53), and the direct effect was −0.01 food groups (95% CI −0.17 to 0.15) out of the total effect estimate of 0.42 food groups (95% CI 0.24 to 0.60). Due to the opposite direction of the indirect and direct effects, the total indirect effect is 102% of the total effect. Garden production was the main pathway by which the intervention increased DDS (*β* = 0.33), accounting for 78% of the intervention’s impact. This pathway operated mainly through increased garden crop species richness, which accounted for a 0.23 increase in DDS, and through improved garden practices, which accounted for a 0.09 increase in DDS. There was also strong evidence for a pathway through crop species richness and market activity, accounting for a 0.02 food group increase in DDS. Poultry production, reflecting the combined pathways of the number of poultry owned, poultry eggs produced and market activity through those indicators, contributed a 0.02 increase in DDS (95% CI 0.004 to 0.03), accounting for only 4% of the intervention effect. The pathways through nutrition knowledge accounted for 18% of the total effect, by contributing a 0.08 increase in DDS (95% CI 0.03 to 0.12), including the indirect effects through knowledge alone (*β* = 0.07) and via market activity (*β* = 0.01). Women’s market activity, including the pathways through production and knowledge, accounted for a 0.03 increase in DDS (95% CI 0.01 to 0.05), corresponding to 7% of the total effect, with evidence only for the sum of the *d* paths via mediators and market activity, but not for the path through market activity alone. The indirect effect of market activity operated through women buying goods, including going to the market to purchase them, with no effect through women’s sale of goods (Supplementary Section 4).

The mediation model results for the intervention’s impact on the proportion of times women met MDD were similar to the results for DDS, and the indirect effects also accounted for all of the total intervention effect (Supplementary Sections 5 and 6). Garden production accounted for three-quarters of the total effect on MDD (*β* = 0.09, that is, a 9% increase in the proportion of times women met MDD). Nutrition knowledge and market activity contributed slightly more to the total effect than in the DDS model (MDD: 22% and 8%, DDS: 18% and 7%), while the indirect effect through poultry production was still negligible (3%).

Heterogeneity analyses of the impact pathways on women’s DDS showed that there were differences by household religion and women’s education level, but not by household wealth (Supplementary Sections 7–9). The indirect effect through women’s market activity was stronger in Muslim women (*β* = 0.05, 9% of the total effect) than in Hindu women (*β* = 0.01, 2% of the total effect). Among women with lower education, home gardening explained 83% of the total effect, compared with 75% in women with at least partial secondary education.

An exploratory mediation analysis that included several indicators of women’s empowerment at endline showed no evidence that increases in women’s social support, communication, decision-making, self-efficacy, mobility, women’s own income or decision-making on own income contributed to the intervention’s impact on DDS, while there was marginal evidence that the strength of a woman’s network may have a small indirect effect (*β* = 0.01, 95% CI −0.002 to 0.03) on DDS (Supplementary Sections 10 and 11). Market activity, which encompasses aspects of women’s empowerment, retained its positive indirect effect, as in other models.

Results of a sensitivity analysis excluding observations during Ramadan were largely consistent with the main model (Supplementary Section 12).

## Discussion

We analysed the impact pathways of the FAARM cluster-randomized trial intervention on women’s dietary diversity in Bangladesh. The intervention was a 3-year, nutrition-sensitive HFP programme encompassing training in home gardening, poultry production and nutrition. We found that increased home garden production accounted for over three-quarters of the intervention’s positive impact on women’s dietary diversity and greater nutrition knowledge for 18%, while the contributions of poultry production and women’s market activity were much smaller.

Our study contributes to the literature on HFP programmes, and complex nutrition-sensitive interventions in general, in a number of ways. It assesses multiple impact pathways on women’s dietary diversity within a single mediation model, using high-frequency panel data from a large-scale, multiyear randomized controlled trial (RCT). Having data from endline as well as surveillance, collected during the intervention and up to 1 year post-intervention, enabled us to aggregate data from several measures for dietary, garden and poultry indicators, which is more robust than a single measure in time. We also had a sample size of over 2,500 participants in 96 settlements, which conferred sufficient power to test multiple mediation pathways.

Our findings show that the HFP intervention achieved impacts on dietary diversity largely as hypothesized in the theory of change, but the contribution of the individual intervention components was far from equal. The paramount importance of home gardening for women’s dietary diversity, in terms of both effect size and strength of evidence, is an important result. While our analyses show that the impact pathways differed slightly by women’s education level and religion, home garden production explained the majority of the intervention’s impact on women’s DDS across all groups.

This large contribution of home gardening is in line with our previous findings that the intervention (1) increased several promoted gardening practices, including fencing, raised beds, pit crops, intercropping, composting, sack gardening, seed storage, cultivation of ‘drought-resistant’ and ‘flood-resistant’ crops, and integrated pest management^35^, (2) more than doubled the number of harvested garden crop species, including dark leafy greens, vitamin-A-rich fruits and vegetables, other vegetables and fruits, with an overall increase by six crop species over the control arm in the final year^35^, and (3) also increased women’s consumption of these food groups^34^. We find that the effect operates primarily through increased crop species richness and not through women’s market activities. These results align with the HFP programme’s intention to grow crops for households’ own consumption, and with our observations that the majority of home gardens were not very large and few produced sufficient surplus to sell. Thus, we conclude that the effect of garden production was primarily through consumption from own production. Several HFP interventions have shown positive impacts on homestead production of diverse foods primarily used for own consumption, with most showing at least modest effects on household or women’s dietary diversity^14,21,22,37^. Importantly, our study provides direct evidence of the pathway linking an HFP programme, agricultural production and dietary diversity through multiple mediation analysis, showing that agricultural diversification is a key mechanism of impact for improving diets.

Poultry production, by contrast, hardly contributed to increasing dietary diversity, adding just 0.02 food groups, corresponding to 4% of the total impact. Although the intervention improved poultry rearing, its impacts were probably too small in size to enable substantial dietary changes (for example, only one-third of intervention households produced eggs)^24^. Selling poultry products can provide an avenue for dietary diversification, and we found evidence that the number of poultry owned led to a very small increase in dietary diversity via women’s market activity (around 0.5% of the intervention effect). Our results reflect documented challenges of poultry production in low-income settings, including disease control and vaccination, predation, theft and proper management^38^, and are in line with the inconsistent impact of poultry rearing on diets reported in the literature^5,16,39,40^.

Women’s nutrition knowledge contributed 18% of the total effect on dietary diversity, an increase of 0.08 food groups. Nutrition counselling is a component of most HFP interventions to ensure that increased agricultural production benefits women and children^5,9^. Interestingly, knowledge on diet diversity contributed directly to improving women’s diets (13% of the total effect), while knowledge on the health function of different food groups contributed through women’s market activity (1% of the total effect). This suggests that the intervention effect on DDS through knowledge was primarily through knowing what foods to eat and the importance of a diverse diet, but that greater nutrition knowledge also enabled women to make informed choices about purchases that supported dietary quality. It is possible that we would have observed an even stronger indirect effect through knowledge had we measured nutrition knowledge at the peak of the intervention (in year 3) rather than when the intervention had ended or at endline (1 year post-intervention), although we assume that women retain the knowledge gained.

Women’s market activity mediated 7% of the increase in women’s dietary diversity. There was strong evidence for market pathways mediated via production and knowledge, but not for women’s market activity directly. This indicator predominantly captured increases in purchasing behaviour, rather than sales. Thus, our findings may reflect the benefits of incorporating market training into the HFP curriculum, as well as the combination of nutrition knowledge (to purchase nutritious foods) with greater agency to decide on purchases. The pathway from production via market may also reflect women’s ability to buy foods, such as eggs, as a result of not needing to purchase vegetables that could be sourced from their gardens. As there was no evidence for pathways via women selling products, these results further support our conclusion that diversified production improved dietary diversity through consumption of own products rather than through women’s income generation.

While aspects of empowerment were implicit in several components of the FAARM intervention and explicit in group activities that focused on intrahousehold allocation, there was no gender- or empowerment-specific curriculum. Exploratory analysis did not provide evidence that increased empowerment overall contributed to the impact on diets, although having stronger networks (more relationships, topics discussed with and support available from other women nearby) emerged as a possible, weak indirect pathway, and may reflect benefits from the intervention’s focus on building women’s farmer groups.

We were able to explain all of the total intervention effect on women’s dietary diversity. This suggests that we probably captured the key domains by which the intervention impacted dietary diversity within our multiple mediation model. Previous evaluations of the FAARM trial found improvements across all of the indicators analysed in this study, which we confirm in our analysis (that is, the ‘*a*’ paths), and through multiple mediation analysis, we could identify home gardening as the most important pathway to improving diets in our study context, and nutrition education as the second most important. Many studies use increases in outputs as evidence for impacts on diets; however, documenting the ‘*a*’ paths but not the ‘*b*’ paths to the outcome can only support the plausibility of the pathways and does not constitute causal evidence. Here, we show that home garden production and nutrition education had direct, causal paths to improved women’s dietary diversity. Understanding the relative importance of intervention impact pathways can help improve the effectiveness of programmes by prioritizing components that have the strongest impacts. For example, we found very limited impact of the HFP poultry component—one of the costliest intervention components—on women’s dietary diversity, suggesting that the poultry component needs to be either improved or dropped in some contexts. Furthermore, considering that women had very limited mobility and purchasing power in our study context, we show that the HFP programme’s focus on diversifying food production for own consumption was an effective strategy in this setting.

Future complex interventions should also consider incorporating multiple mediation analyses to establish evidence of PIPs. It is essential for mediation analyses to be considered at the design stage to collect appropriate data for indicators during the intervention. While we did consider mediation in our study design, there are still limitations concerning aspects of data collection and analysis. First, we chose a simpler over a more complex model to ensure interpretability and model feasibility by limiting the number of indicators for each domain, and using easy-to-interpret variables rather than indices or latent variables. We assume reasonable validity of the chosen indicators, but they may not fully capture the intended domains, which could have influenced the strength of the relative pathways. In particular, nutrition knowledge was captured at two timepoints at the end of the trial, with one indicator measured about 1 year post-intervention, and market activity was also captured at only one timepoint towards the end of the intervention, in comparison with the more robust garden production indicator, which relied on data measured frequently throughout the trial. Second, given the measurement of different indicators at different timepoints, we made certain assumptions to align with causal mediation requirements of temporality, as described in the Methods. We minimized the number of pathways between variables, particularly when bidirectional relationships or temporal violations were possible (for example, between garden practices and crop species richness). Third, we could not capture cyclical effects between the mediators (for example, increased garden production improving diets or purchasing power, further motivating increased garden production), as we used averages to assess impacts over a longer time frame. Fourth, because the trial was powered for impacts on the primary outcome, and not powered a priori for mediation analysis, we have discussed uncertainty in our estimates throughout. Fifth, while both promoted and non-promoted crops increased, we cannot rule out the possibility of trade-offs between crop production and poultry, as well as other livestock, fish or animal products. As we do not have data on the production of animal species and animal products during the intervention, but only post-intervention, these types of trade-offs could not be explored. Finally, we did not examine household income as a mediator owing to data limitations. However, we found no mediating effects through women’s sale of goods or women’s own income, and the majority of households did not sell surplus garden products, suggesting that household income would not be a main impact pathway. Nevertheless, it is possible that there are other indirect pathways, synergies and trade-offs of the FAARM intervention’s impact on women’s dietary diversity that we could not explore due to data limitations, such as impacts on other household members’ farm and non-farm activities, income and market activity.

There are also general limitations to the interpretation of our findings. First, they are specific to the context of rural Sylhet in northeast Bangladesh, where women traditionally have limited mobility and market access, making our findings most generalizable to HFP programmes in similar settings. We also focus on only one outcome of the FAARM trial, women’s dietary diversity. It is possible that components of the FAARM intervention that played a limited role in improving women’s diets (for example, poultry) may have stronger indirect effects on other outcomes. Lastly, while this mediation analysis can tell us which pathways were most important to achieving impacts, a process evaluation would be needed to better understand the barriers and facilitators affecting these pathways that need to be addressed to ensure optimal impacts on outcomes^41^. For example, further quantitative and qualitative research could provide insight into whether the negligible indirect effect through poultry was due to implementation challenges, common poultry challenges such as disease, or appropriateness and acceptability of poultry in our study context.

In conclusion, our study addresses an imperative to disentangle the impact pathways of complex interventions, doing so through robust mediation analysis of a large-scale, multiyear HFP trial in Bangladesh. Our findings show that the FAARM intervention improved women’s dietary diversity primarily through increased consumption of harvested garden crops, and that nutrition knowledge also made an important contribution, while women’s market activity played a smaller role. Poultry production accounted for the smallest share of the intervention’s impact on women’s diets, and there was little evidence of an impact pathway through other indicators of women’s empowerment beyond marketing. Our findings can be used to guide the development of future interventions, and point to focusing on home garden diversification as an important lever for improving diets. Conducting multiple mediation analysis across a range of nutrition-sensitive agriculture interventions and synthesizing the evidence on key impact pathways will be crucial to achieving nutrition and health goals in low- and middle-income countries.

## Methods

### Study design

FAARM was a cluster RCT conducted in Habiganj district, Sylhet Division, Bangladesh, from 2015 to 2020. The study aimed to evaluate the impact of an HFP programme on the nutrition and health of women and their children. The intervention was conducted at the cluster level to facilitate delivery through woman farmer groups and enable separation of the two trial arms. The FAARM trial protocol provides details on the study design and methods^19^.

The FAARM trial was positively reviewed by the ethics committees of the Medical Faculty at Heidelberg University in Germany and the James P Grant School of Public Health at BRAC University in Bangladesh. All FAARM participants provided written, informed consent.

### Participants

The trial’s study population consisted of women and their children up to approximately 3 years of age. Women were eligible if their self-reported age was 30 years or younger, they were married, they had access to at least 40 m^2^ of land and they were interested in participating in an HFP programme.

### Randomization and masking

Before conducting the baseline survey, eligible women gave their consent and were enrolled. After the baseline survey, 96 settlements (geographic clusters of households separated by a 400-m buffer) were randomized 1:1 to the control and intervention group using covariate-constrained randomization^42^. Participants were not informed of their assignment. Data collectors were masked to intervention assignment and were separate from intervention staff, although intervention activities may have been observed. To enable project learning and adjustment, data analysts were not masked to intervention assignment.

### Procedures

The non-governmental organization Helen Keller International implemented the HFP programme from mid-2015 to late 2018. The programme included training in home gardening, poultry rearing, nutrition and hygiene. Training was provided to women and their families through group sessions. Project staff also visited households and conducted individual counselling every 2 months to provide technical advice on gardening and poultry rearing, a review of nutrition topics tailored to the family, and the opportunity to discuss problems. Attendance at training sessions was high, ranging from 68% to 85% per session. Further description of the intervention components can be found in Supplementary Section 13 and the study protocol^19^.

Data were collected on tablets by trained data collection officers using face-to-face interviews. The baseline survey was conducted from March to May 2015. A surveillance system was implemented to collect data every 2 months from September 2015 to August 2019. As part of the surveillance system’s routine assessment component, women’s dietary diversity data were collected every 6 months (with a rotating one-third of women interviewed every 2 months), garden production data every 4 months, and poultry production data annually. The endline survey was administered from September 2019 to April 2020. For this analysis, we used available data from the latter half of the trial, when intervention activities should have reached full impact (from mid-2017)^34^. A timeline of intervention activities and data collection periods for the variables used in this analysis is included in Supplementary Section 14.

Dietary diversity was assessed by asking women to freely recall all foods they had consumed in the past 24 h. Enumerators then classified consumed foods into 21 food groups^43^. Women were asked about any food groups not mentioned to verify that no food groups were missed. For this analysis, we used the four last dietary assessments for each woman (March 2018 to February 2020; encompassing data collected every 6 months from the last year and a half of the routine assessment plus the endline survey). In a sensitivity analysis, we excluded surveys that occurred during Ramadan because of changes in women’s dietary diversity during this period^44^.

To assess garden production, women were asked to report the crop species they had harvested in the previous season. We used data from the latter five data collection points (March 2018 to September 2019). As women were asked to report harvested crop species in the previous season, the recall period preceded that of the dietary assessments and, by using an average over time, should represent women’s usual garden production in the second half of the intervention. In 2018, data on garden elements and practices (for example, raised beds, intercropping and organic fertilizer) were collected during walk-throughs of the garden.

The poultry production and management assessment included questions on adult chicken and duck ownership, poultry egg production, vaccination practices, and shed ownership and use. As with garden production, we use the latter two annual surveys conducted in 2018 and 2019 to estimate women’s usual poultry production in the latter half of the intervention, and to capture production that temporally precedes dietary consumption.

To evaluate women’s nutrition knowledge, we used data from questions asked about diet diversity knowledge during the routine assessment (November to December 2018) as well as knowledge questions about the benefits of food groups at endline (January to March 2020). The latter assessment had to stop early due to the COVID-19 lockdown in March 2020, resulting in a lower sample size.

In the last rounds of routine assessment (March to August 2019), women were asked about market-related activities, including whether women bought and sold goods and whether women went to market to buy goods. Questions about women’s empowerment were asked at baseline as well as from March to December 2019.

### Variables

All variables included in the analysis are described in detail in Supplementary Section 15. The main outcome variable was women’s average DDS, calculated as the sum of ten food groups consumed in the prior 24 h (ref. ^43^), averaged over up to four dietary assessment rounds. A second outcome variable was the proportion of times a woman met MDD (≥5 out of 10 food groups), calculated as the number of times MDD was met divided by the number of data collection rounds (1–4). Among the 2,612 women with dietary data in the selected time period, 75% had data from all four rounds, 12% from three rounds, 5% from two rounds and 8% from one round. When excluding observations that occurred during Ramadan, for the sensitivity analysis, 56% of women had data from all four rounds, 26% from three rounds, 10% from two rounds and 8% from one round.

The mediators included indicators to capture home gardening (crop species richness and garden practices score), poultry production (number of poultry owned and poultry eggs produced), women’s nutrition knowledge (diet diversity knowledge score and food group knowledge score) and women’s market activity (market score). Garden crop species richness was calculated as the total number of crop species harvested in the home garden in the prior season, averaged over up to five assessment rounds. Gardening practices were captured as a sum score of the number of promoted garden practices applied. Poultry ownership was calculated as the average number of adult ducks and chickens owned, and the production of poultry eggs was calculated as the average number of eggs produced in the prior week, both averaged over up to two annual surveys.

To assess diet diversity knowledge, women were asked to name the types of food an adult woman should eat regularly, and were also asked for reasons why a diverse diet is important during pregnancy and lactation. Food group knowledge was measured using a score based on the number of correct answers to five multiple-choice questions about the health function of certain food groups, reflecting information taught in nutrition sessions. These data were collected post-intervention; however, we make the assumption that we are capturing nutrition knowledge that would have been gained temporally before the dietary assessments.

Market activity was captured using a sum score of whether the woman had, in the last month, bought goods, sold goods, or gone to market to buy goods. We interpret the market activity variable as an indicator of women’s engagement with markets, for example, through mobile vendors, not per se physically going to the market themselves, as less than one-quarter of women physically went to the market in the last month to buy goods, and among those who sold goods, almost all did so to neighbours or through intermediaries or family members. We also expect the market score to capture some aspects of women’s empowerment in the context of our study, including intrinsic and instrumental agency.

In a supplementary analysis, we also explore pathways through other indicators of women’s empowerment measured at endline, including social support, communication with husband, external communication, decision-making, self-efficacy, network, mobility, women’s own income and decision-making on her income.

Several baseline covariates were included in the analysis as potential mediator–outcome confounders and to increase precision: women’s DDS at baseline, household wealth quintile at baseline (based on household assets), household religion (Muslim or Hindu), homestead and agricultural land size, garden crop species richness at baseline, women’s market score at baseline, women’s education level (completed school years) and indicators of women’s empowerment at baseline, including mobility score, social support score, communication with husband score, external communication score, own income and decision-making score.

### Statistical analysis

The FAARM trial is registered with ClinicalTrials.gov (ID: NCT025-05711). The study sample size was calculated based on the trial’s primary outcome, child length/height-for-age *z* score. No additional sample size calculations were conducted for the mediation analysis.

To assess the intervention’s total effect on the outcome and mediators, we conducted mixed-effects regression analyses, including random effects at the settlement level.

We conducted causal mediation analyses using structural equation models to assess the direct and indirect effects of the intervention on dietary diversity based on a DAG (Fig. 1). The model specified direct paths from the intervention to the outcome and mediators. Women’s market activity was also regressed on the garden, poultry and knowledge mediators to include the indirect paths through market score. The model controlled for hypothesized mediator–outcome confounders, selected a priori based on theory. Within the SEM, we allowed for correlations between the residuals of all the poultry and garden mediators and between the two knowledge mediators. We decomposed the total effect (the effect of the intervention on dietary diversity) into indirect effects (the effects of the intervention on dietary diversity through each mediator), specific indirect effects (the effects of the intervention on dietary diversity through each mediator and market activity) and the direct effect (the remaining effect of the intervention on dietary diversity that is not explained by the mediators). Indirect effects were then summed to capture the overall indirect effects through each mediator domain, namely, through garden, poultry and knowledge (with and without paths through market activity), and the indirect effect of market activity.

We estimated the model using full information maximum likelihood to account for missing data, a method that allowed us to use all available data from all analysed participants in our estimation, under the assumption that data were missing at random. Our estimation also accounted for clustering by settlement. Monte Carlo CIs were calculated with 10,000 repetitions. Our model included 2,612 women and 204 parameters (approximately 13 observations per parameter), providing a sufficient sample size for estimation. We evaluated model fit using the chi-square test (*P* > 0.05) and standard thresholds for CFI (≥0.95), TLI (≥0.95) and RMSEA (≤0.06)^45^.

We conducted these analyses for two outcomes: average DDS and the proportion of times MDD was met. As a sensitivity analysis, we conducted the analysis for DDS removing observations that occurred during Ramadan. We also conducted heterogeneity analyses, stratifying the main analysis by women’s religion (Muslim; Hindu), household wealth (low = quintiles 1, 2; high = quintiles 3, 4, 5), and women’s education level (low = no or partial/complete primary education; high = partial or complete secondary education). To explore pathways through women’s market activity, we also disaggregated the market score into its components (that is, buying goods, selling goods, and going to market to buy goods) and analysed indirect effects through the production and knowledge mediators, as well as these market variables.

We conducted exploratory mediation analysis with indicators of women’s empowerment to determine whether empowerment had an indirect effect on DDS independent of women’s market activity. We mapped all indirect paths without additional specific indirect pathways through marketing to assess these independent effects, and included residual correlations between the empowerment mediators.

Given the RCT design, the assumption of no unmeasured confounding of the exposure–mediator and exposure–outcome relationship holds. In addition, we assume no unmeasured confounding of the mediator–outcome relationships, that the regressions are linear—which allows us to add the indirect effects—and that there are no exposure–mediator interactions. We also rely on the assumptions that our measures are reliable and that the included variables follow the temporal paths shown in the DAG to minimize the possibility of reverse causality.

Data processing and analyses were conducted using Stata MP version 18.0 (StataCorp). Structural equation models were fitted in R (version 4.5.0) using the ‘lavaan’ package (0.6-19)^46^, and Monte Carlo 95% CIs were calculated using semTools (0.5-7)^47^. We followed guidelines from the AGReMA^48^ statement and CONSORT^49^.

### Reporting summary

Further information on research design is available in the Nature Portfolio Reporting Summary linked to this article.

## Supplementary information

Supplementary Information Supplementary Sections 1–15.

Reporting Summary

## Data Availability

The aggregated dataset for the replication of this study is available via GitHub at https://github.com/nathaliejlambrecht/FAARM-impactpathwaysDDS-analysis. A deidentified dataset with the individual participant response data that underlie the results reported in this article is available upon request. Interested researchers will need to provide a methodologically sound proposal for use of the data and sign a data access agreement to gain access to the data. The underlying individual response data are not publicly available due to privacy restrictions and their complexity. Data requests with a proposal should be directed to the corresponding author (N.J.L.) and the principal investigator (S.G.). The FAARM trial protocol is available online.

## Code availability

The code for replication of this study is available via GitHub at https://github.com/nathaliejlambrecht/FAARM-impactpathwaysDDS-analysis.
