# Supplementary material for: Impact pathways of a homestead food production programme on women’s dietary diversity in Bangladesh
Source: Nat Food. 2026 May 12;7(5):464–73. doi: 10.1038/s43016-026-01354-9 (PMC13212147; doi:10.1038/s43016-026-01354-9)
Supplement: Supplementary file 1 — Supplementary Sections 1–15. [file 43016_2026_1354_MOESM1_ESM.pdf]

nature food

Supplementary information https://doi.org/10.1038/s43016-026-01354-9

Impact pathways of a homestead food production programme on women's dietary diversity in Bangladesh

In the format provided by the authors and unedited

Impact pathways of a homestead food production programme on women's dietary diversity in Bangladesh

Lambrecht et al.

Supplementary Information

Supplementary Information 1. Trial profile and analytic sample selection. 2
Supplementary Information 2. Causal mediation pathways of the FAARM intervention on women's dietary diversity score with standardized estimates. 3
Supplementary Information 3. Estimates and p-values for path coefficients of the mediation pathways of the FAARM intervention on women's dietary diversity score. 4
Supplementary Information 4. Direct and indirect effects of the FAARM intervention on women's dietary diversity score through garden production, poultry production, nutrition knowledge, and separate market activity mediators. 5
Supplementary Information 5. Causal mediation pathways of the FAARM intervention on women's minimum dietary diversity. 6
Supplementary Information 6. Direct and indirect effects of the FAARM intervention on the proportion of times women met minimum dietary diversity. 7
Supplementary Information 7. Heterogeneity in direct and indirect effects of the FAARM intervention on women's dietary diversity score, by religion. 8
Supplementary Information 8. Heterogeneity in direct and indirect effects of the FAARM intervention on women's dietary diversity score, by wealth. 9
Supplementary Information 9. Heterogeneity in direct and indirect effects of the FAARM intervention on women's dietary diversity score, by women's education level. 10
Supplementary Information 10. Causal mediation pathways of the FAARM intervention on women's dietary diversity score through women's home garden production, poultry production, nutrition knowledge, market activity, and empowerment. 11
Supplementary Information 11. Direct and indirect effects of the FAARM intervention on women's dietary diversity score through women's home garden production, poultry production, nutrition knowledge, market activity, and empowerment. 12
Supplementary Information 12. Direct and indirect effects of the FAARM intervention on women's dietary diversity score, excluding women with observations during Ramadan. 13
Supplementary Information 13. Supplementary methods. 14
Supplementary Information 14. Timeline of intervention activities. 15
Supplementary Information 15. Variables included in the mediation analysis. 16

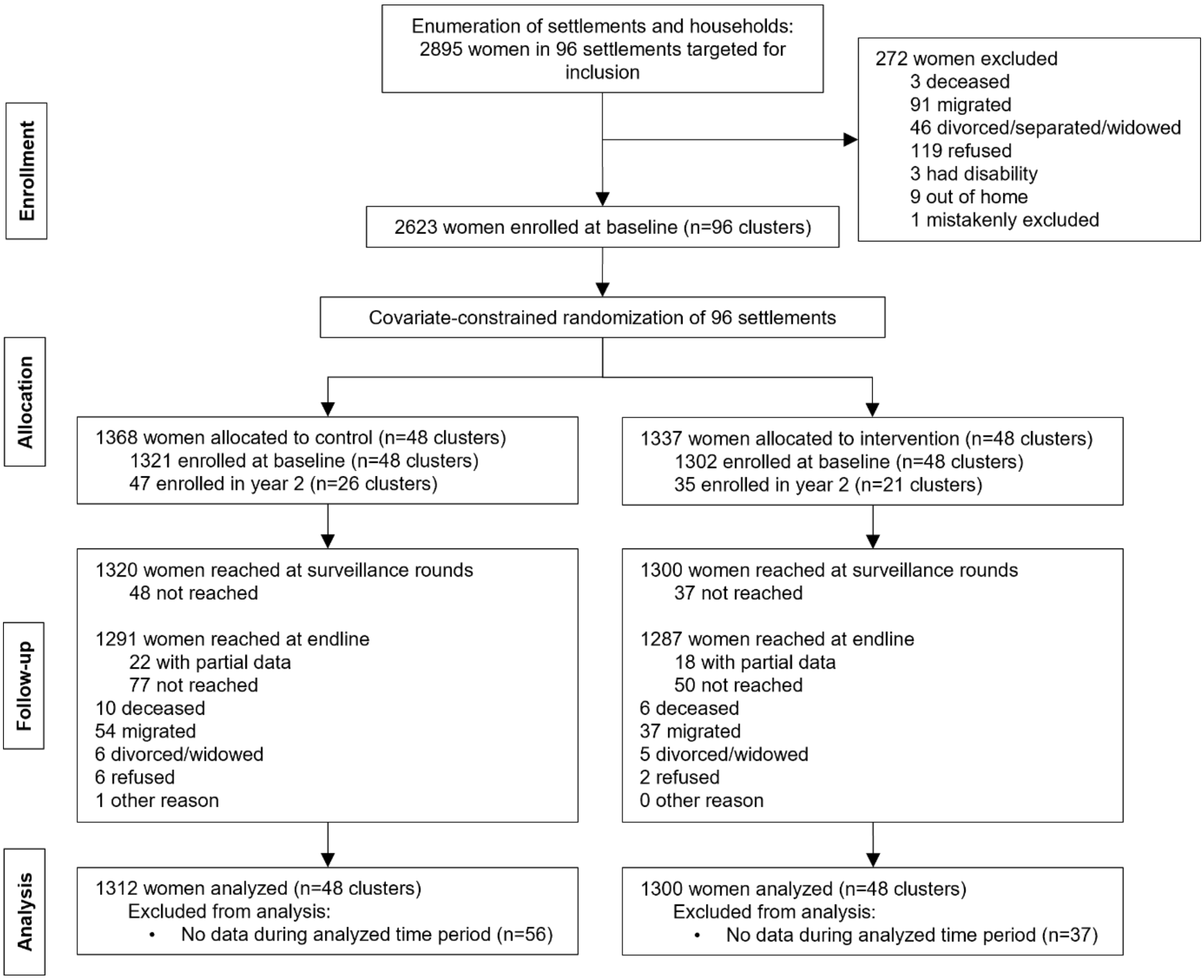

Supplementary Information 1. Trial profile and analytic sample selection.
Women were enrolled before randomization. In year 2, women who were newly married into enrolled households were recruited for participation.

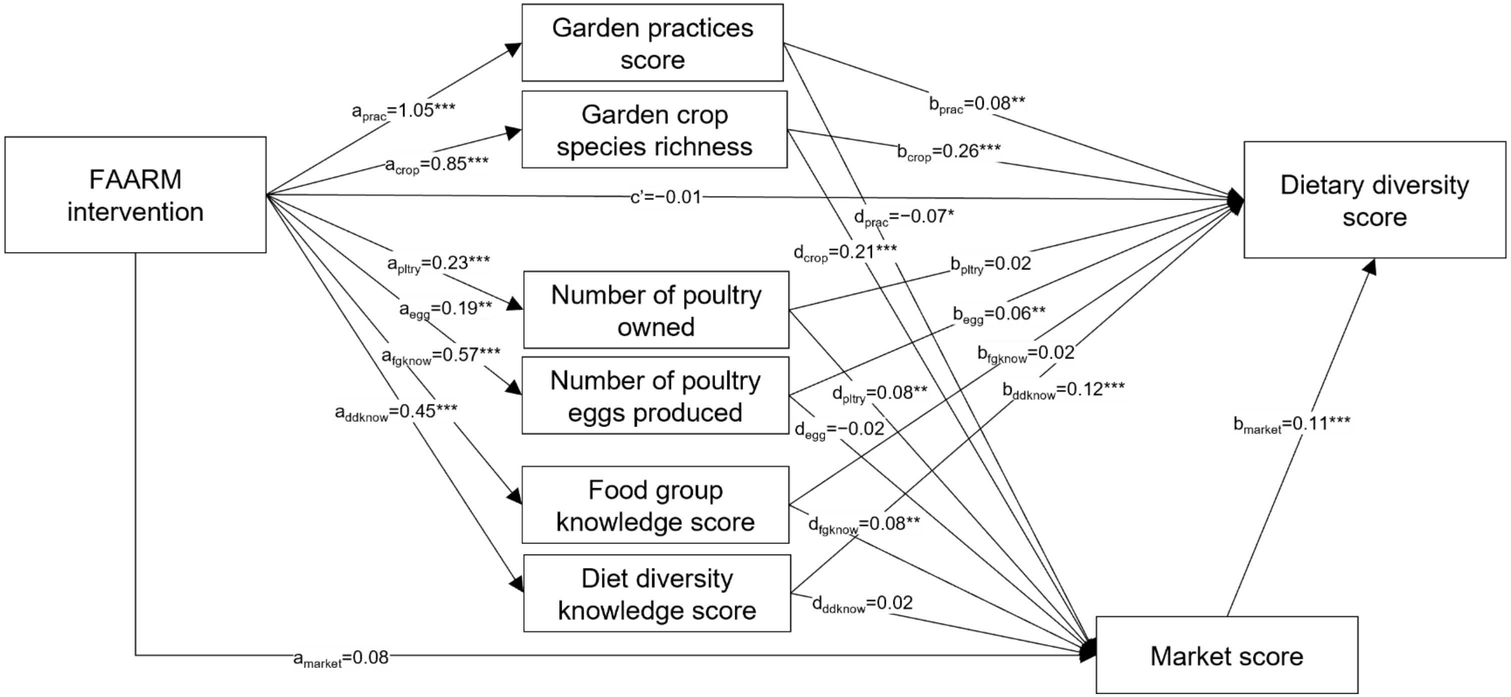

Supplementary Information 2. Causal mediation pathways of the FAARM intervention on women's dietary diversity score with standardized estimates.

Coefficients of the *a* paths represent the effect of the (unstandardized) intervention (coded 1 for intervention, 0 for control) on the mediator in standard deviation units of the mediator. Coefficients of the *b* paths represent the effect of a one standard deviation increase in the mediator on the women's dietary diversity score, and coefficients of the *d* paths represent the effect of a one standard deviation increase in the mediator on women's market score. Baseline covariates were not standardized. Residual correlations and variances are omitted from the figure. P-values were estimated using unstandardized variables: *p<0.05, **p<0.01, ***p<0.001

Supplementary Information 3. Estimates and p-values for path coefficients of the mediation pathways of the FAARM intervention on women's dietary diversity score, as shown in Figure 2. Path coefficients estimated using structural equation modeling (n=2,612) with Full Information Maximum Likelihood and cluster-adjusted standard errors, controlling for baseline covariates. P-values are estimated using two-sided Wald tests.

**Path**	**β**	**95% CI**	**p-value**
a_{pltry}	0.926	0.416, 1.440	<0.001
a_{egg}	1.545	0.511, 2.563	0.003
a_{prac}	4.541	3.677, 5.416	<0.001
a_{crop}	4.983	4.108, 5.865	<0.001
a_{fgknow}	0.612	0.481, 0.744	<0.001
a_{ddknow}	0.398	0.231, 0.569	<0.001
a_{market}	0.072	-0.046, 0.194	0.23
b_{pltry}	0.005	-0.009, 0.019	0.50
b_{egg}	0.008	0.002, 0.013	0.01
b_{prac}	0.020	0.005, 0.036	0.009
b_{crop}	0.045	0.035, 0.056	<0.001
b_{fgknow}	0.024	-0.023, 0.070	0.32
b_{ddknow}	0.140	0.079, 0.201	<0.001
b_{market}	0.127	0.080, 0.173	<0.001
d_{pltry}	0.019	0.005, 0.033	0.009
d_{egg}	-0.003	-0.010, 0.005	0.48
d_{prac}	-0.015	-0.029, -0.002	0.03
d_{crop}	0.033	0.021, 0.044	<0.001
d_{fgknow}	0.066	0.021, 0.112	0.005
d_{ddknow}	0.021	-0.024, 0.068	0.36

Supplementary Information 4. Direct and indirect effects of the FAARM intervention on women's dietary diversity score through garden production, poultry production, nutrition knowledge, and separate market activity mediators.

	**β**	**95% CI**
Total effect	0.423	0.240, 0.603
Direct effect	-0.011	-0.172, 0.149
Indirect effect	0.434	0.338, 0.535
Indirect effects through market[a]		
Garden practices score		
→ Bought goods → DDS	-0.003	-0.010, 0.001
→ Bought goods at market → DDS	-0.005	-0.013, 0.001
→ Sold goods → DDS	-0.001	-0.004, 0.001
Garden crop species richness		
→ Bought goods → DDS	0.007	0.000, 0.017
→ Bought goods at market → DDS	0.009	0.002, 0.018
→ Sold goods → DDS	0.003	-0.003, 0.010
Number of poultry owned		
→ Bought goods → DDS	0.000	0.000, 0.001
→ Bought goods at market → DDS	0.001	0.000, 0.002
→ Sold goods → DDS	0.001	-0.001, 0.002
Number of poultry eggs produced		
→ Bought goods → DDS	-0.001	-0.002, 0.000
→ Bought goods at market → DDS	-0.001	-0.002, 0.000
→ Sold goods → DDS	0.000	0.000, 0.001
Food group knowledge score		
→ Bought goods → DDS	0.002	0.000, 0.005
→ Bought goods at market → DDS	0.003	0.000, 0.007
→ Sold goods → DDS	0.000	-0.001, 0.002
Diet diversity knowledge score		
→ Bought goods → DDS	0.000	-0.001, 0.001
→ Bought goods at market → DDS	0.001	0.000, 0.004
→ Sold goods → DDS	0.000	0.000, 0.001
Indirect effects through market alone[b]*:*		
Bought goods → DDS	0.006	-0.001, 0.018
Bought goods at market → DDS	0.005	-0.005, 0.017
Sold goods → DDS	-0.001	-0.005, 0.002
Indirect effects, independent of market[c]*:*		
Garden practices score → DDS	0.092	0.024, 0.165
Garden crop species richness → DDS	0.228	0.160, 0.304
Number of poultry owned → DDS	0.005	-0.008, 0.018
Number of poultry eggs produced → DDS	0.012	0.002, 0.026
Food group knowledge score → DDS	0.015	-0.015, 0.042
Diet diversity knowledge score → DDS	0.055	0.026, 0.092

Note: Estimates are unstandardized effects with 95% Monte Carlo confidence intervals. The structural equation model controlled for the following baseline covariates as potential mediator-outcome confounders: religion, household wealth quintile, log-transformed homestead land size, log-transformed agricultural land size, garden crop species richness, market score, women's education, domains of women's empowerment (including mobility, social support, communication with husband, external communication, own income, and decision-making), and women's dietary diversity score. We estimated the model using Full Information Maximum Likelihood (FIML). We allowed for correlations between the residuals of all the poultry and garden mediators, between the residuals of the knowledge mediators, and between the residuals of the market mediators. The model includes 2612 women. Fit statistics: CFI=0.994; TLI=0.822; RMSEA=0.045; $\chi^2(8)=49.82$, $p<0.001$

[a]Indirect effects are calculated as a×d×b for the production/knowledge (a, b paths) and market mediators (d paths) (e.g., $a_{crop\_species\_richness} \times d_{buy\_goods} \times b_{crop\_species\_richness}$).

[b]Indirect effects are calculated as a×b for the market mediators (e.g., $a_{buy\_goods} \times b_{buy\_goods}$).

[c]Indirect effects are calculated as a×b for the production/knowledge mediators (e.g., $a_{crop\_species\_richness} \times b_{crop\_species\_richness}$).

Abbreviations: CFI – Comparative Fit Index, CI – confidence interval, RMSEA – Root Mean Square Error of Approximation, TLI – Tucker-Lewis Index

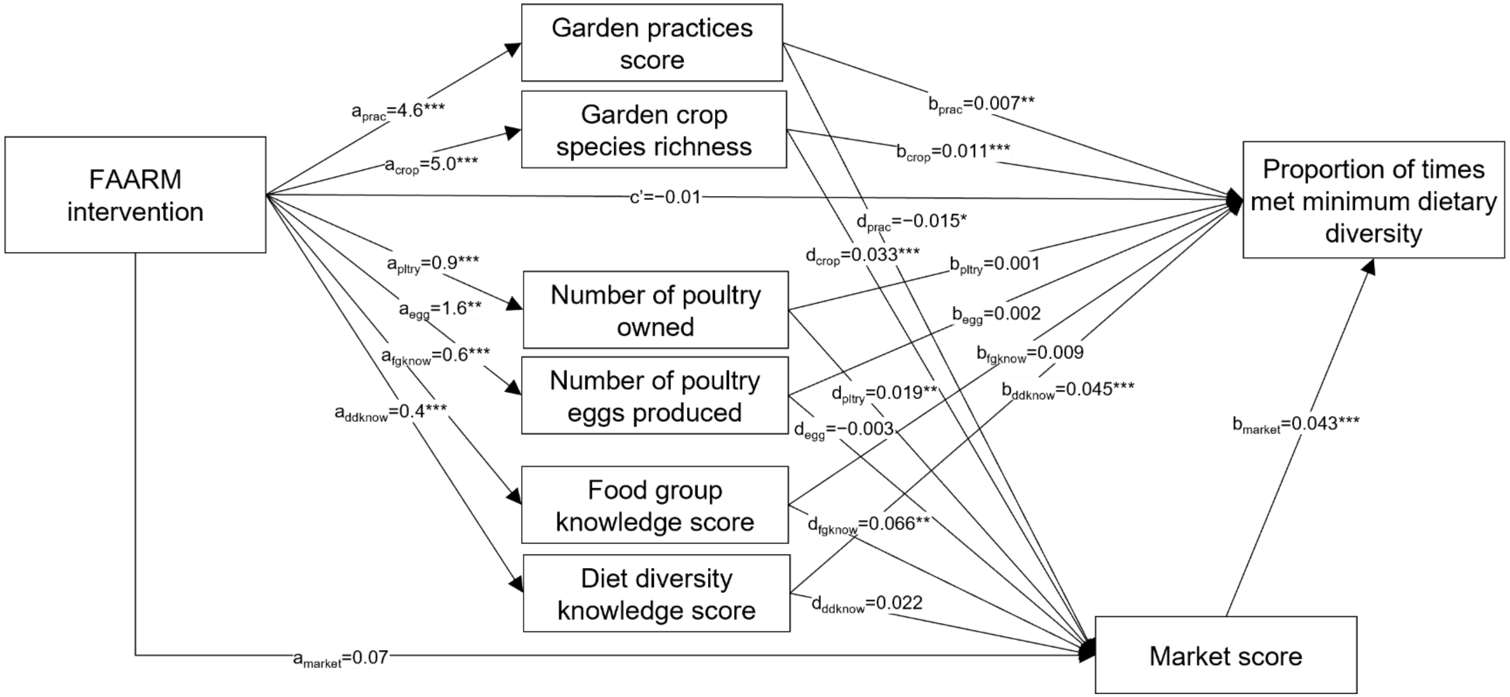

Supplementary Information 5. Causal mediation pathways of the FAARM intervention on women's minimum dietary diversity.
Path coefficients estimated using structural equation modeling (n=2,612) with Full Information Maximum Likelihood and cluster-adjusted standard errors, controlling for baseline covariates. Unstandardized coefficients of the *a* paths are the effect of the intervention on the mediators, coefficients of the *b* paths are the effect of a one-unit increase in the mediators on the proportion of times women met dietary diversity, and coefficients of the *d* paths are the effect of a one-unit increase in the mediators on women's market score. Residual correlations and variances are omitted from the figure. Corresponding indirect effects are included in Supplementary Information 6. P-values are estimated using two-sided Wald tests. P-values: *p<0.05, **p<0.01, ***p<0.001

Supplementary Information 6. Direct and indirect effects of the FAARM intervention on the proportion of times women met minimum dietary diversity (as shown in Supplementary Information 5).

	β	95% CI	Proportion mediated
Total effect[a]	0.119	0.066, 0.170	
Direct effect	-0.005	-0.052, 0.042	
Indirect effect[b]	0.123	0.095, 0.154	104.0%
<u>*Individual indirect effects:*</u>			
Home garden production			
Garden practices score (a_{prac}*b_{prac})	0.031	0.007, 0.058	
Garden crop species richness (a_{crop}*b_{crop})	0.055	0.036, 0.075	
Garden practices via market (a_{prac}*d_{prac}*b_{market})	-0.003	-0.006, 0.000	
Garden crops via market (a_{crop}*d_{crop}*b_{market})	0.007	0.004, 0.012	
Poultry production			
No. of poultry owned (a_{pltry}*b_{pltry})	0.001	-0.004, 0.006	
No. of poultry eggs produced (a_{egg}*b_{egg})	0.003	-0.001, 0.007	
Poultry owned via market (a_{pltry}*d_{pltry}*b_{market})	0.001	0.000, 0.002	
Poultry eggs via market (a_{egg}*d_{egg}*b_{market})	0.000	-0.001, 0.000	
Nutrition knowledge			
Food group knowledge score (a_{fgknow}*b_{fgknow})	0.006	-0.005, 0.016	
Diet diversity knowledge score (a_{ddknow}*b_{ddknow})	0.018	0.009, 0.030	
Food group score via market (a_{fgknow}*d_{fgknow}*b_{market})	0.002	0.000, 0.004	
Diet diversity score via market (a_{ddknow}*d_{ddknow}*b_{market})	0.000	0.000, 0.001	
Market activity			
Market score (a_{market}*b_{market})	0.003	-0.002, 0.009	
Production/knowledge via market (∑a*d*b paths)	0.007	0.003, 0.012	
<u>*Indirect effects by domain:*</u>			
Home garden production (∑prac+crop)	0.086	0.061, 0.114	72.6%
Home garden production w/market (∑prac+crop+garden prod via market)	0.090	0.065, 0.119	76.1%
Poultry production (∑pltry+egg)	0.003	0.000, 0.008	2.9%
Poultry production w/market (∑pltry+egg+poultry prod via market)	0.004	0.001, 0.009	3.4%
Nutrition knowledge (∑fgknow+ddknow)	0.024	0.009, 0.039	20.1%
Nutrition knowledge w/market (∑fgknow+ddknow+know via market)	0.026	0.011, 0.042	22.0%
Market activity (∑market+prod/know via market)	0.010	0.004, 0.017	8.4%

Note: Estimates are unstandardized effects with 95% Monte Carlo confidence intervals. The structural equation model controlled for the following baseline covariates as potential mediator-outcome confounders: religion, household wealth quintile, log-transformed homestead land size, log-transformed agricultural land size, garden crop species richness, market score, women's education, domains of women's empowerment (including mobility, social support, communication with husband, external communication, own income, and decision-making), and women's dietary diversity score. We estimated the model using Full Information Maximum Likelihood (FIML) and allowed for correlations between the residuals of all the poultry and garden mediators and the residuals of the knowledge mediators. The model includes 2612 women.
Fit statistics: CFI=0.993; TLI=0.830; RMSEA=0.045; $\chi^2(8)=49.49$, p<0.001
[a]The total effect is the sum of the direct effect and the total indirect effect.
[b]The indirect effect is calculated as the sum of the indirect effects through garden production, poultry production, nutrition knowledge, and marketing activity.
Abbreviations: CFI – Comparative Fit Index, CI – confidence interval, No. - number, RMSEA – Root Mean Square Error of Approximation, TLI – Tucker-Lewis Index, w/ - with

Supplementary Information 7. Heterogeneity in direct and indirect effects of the FAARM intervention on women's dietary diversity score, by religion.

	Group	β	95% CI	Interaction p-value	Proportion mediated
Total effect	Hindu	0.274	-0.033, 0.579		
	Muslim	0.494	0.307, 0.676		
	Difference (M−H)	*0.220*	*-0.137, 0.578*	*0.22*	
Direct effect	Hindu	-0.021	-0.297, 0.256		
	Muslim	-0.007	-0.179, 0.163		
	Difference (M−H)	*0.014*	*-0.312, 0.339*	*0.93*	
Indirect effect	Hindu	0.295	0.156, 0.447		107.8%
	Muslim	0.501	0.387, 0.621		101.5%
	Difference (M−H)	*0.206*	*0.016, 0.394*	*0.03*	
Indirect effects by domain:					
Home garden production	Hindu	0.226	0.122, 0.355		82.6%
	Muslim	0.361	0.262, 0.470		73.1%
	Difference (M−H)	*0.135*	*-0.027, 0.288*	*0.09*	
Home garden production w/market	Hindu	0.231	0.125, 0.364		84.4%
	Muslim	0.376	0.276, 0.485		76.2%
	Difference (M−H)	*0.145*	*-0.019, 0.299*	*0.07*	
Poultry production	Hindu	0.025	-0.011, 0.061		9.1%
	Muslim	0.010	-0.002, 0.025		2.0%
	Difference (M−H)	*-0.015*	*-0.053, 0.025*	*0.41*	
Poultry production w/market	Hindu	0.025	-0.011, 0.061		9.2%
	Muslim	0.012	-0.001, 0.027		2.4%
	Difference (M−H)	*-0.014*	*-0.051, 0.026*	*0.45*	
Nutrition knowledge	Hindu	0.038	-0.015, 0.103		14.1%
	Muslim	0.086	0.033, 0.142		17.5%
	Difference (M−H)	*0.048*	*-0.036, 0.126*	*0.23*	
Nutrition knowledge w/market	Hindu	0.041	-0.013, 0.106		14.8%
	Muslim	0.094	0.042, 0.151		19.1%
	Difference (M−H)	*0.054*	*-0.030, 0.132*	*0.18*	
Market activity	Hindu	0.006	-0.003, 0.024		2.1%
	Muslim	0.044	0.020, 0.073		8.8%
	Difference (M−H)	*0.038*	*0.009, 0.069*	*0.01*	

Note: Estimates are unstandardized effects with 95% Monte Carlo confidence intervals. The structural equation model controlled for the following baseline covariates as potential mediator-outcome confounders: household wealth quintile, log-transformed homestead land size, log-transformed agricultural land size, garden crop species richness, market score, women's education, domains of women's empowerment (including mobility, social support, communication with husband, external communication, own income, and decision-making), and women's dietary diversity score. We estimated the model using Full Information Maximum Likelihood (FIML) and allowed for correlations between the residuals of all the poultry and garden mediators and the residuals of the knowledge mediators. The model includes 2612 women. Fit statistics: CFI=0.994; TLI=0.864; RMSEA=0.041; $\chi^2(16)=51.1$, $p<0.001$. Abbreviations: CFI – Comparative Fit Index, CI – confidence interval, RMSEA – Root Mean Square Error of Approximation, TLI – Tucker-Lewis Index, w/ - with

Supplementary Information 8. Heterogeneity in direct and indirect effects of the FAARM intervention on women's dietary diversity score, by wealth (lower = quintiles 1, 2; higher = quintiles 3, 4, 5).

	**Group**	**β**	**95% CI**	**Interaction p-value**	**Proportion mediated**
Total effect	Lower wealth	0.435	0.196, 0.666		
	Higher wealth	0.421	0.238, 0.602		
	Difference (H−L)	*-0.015*	*-0.311, 0.285*	*0.92*	
Direct effect	Lower wealth	-0.021	-0.240, 0.200		
	Higher wealth	-0.002	-0.178, 0.173		
	Difference (H−L)	*0.019*	*-0.263, 0.299*	*0.90*	
Indirect effect	Lower wealth	0.456	0.336, 0.583		104.8%
	Higher wealth	0.423	0.308, 0.543		100.5%
	Difference (H−L)	*-0.033*	*-0.204, 0.142*	*0.70*	
Indirect effects by domain:					
Home garden production	Lower wealth	0.341	0.240, 0.448		78.2%
	Higher wealth	0.311	0.211, 0.422		73.9%
	Difference (H−L)	*-0.030*	*-0.176, 0.123*	*0.69*	
Home garden production w/market	Lower wealth	0.360	0.256, 0.469		82.6%
	Higher wealth	0.318	0.216, 0.431		75.6%
	Difference (H−L)	*-0.042*	*-0.191, 0.113*	*0.59*	
Poultry production	Lower wealth	0.014	0.001, 0.033		3.2%
	Higher wealth	0.017	-0.002, 0.037		4.1%
	Difference (H−L)	*0.003*	*-0.024, 0.028*	*0.79*	
Poultry production w/market	Lower wealth	0.015	0.001, 0.033		3.3%
	Higher wealth	0.019	0.000, 0.039		4.6%
	Difference (H−L)	*0.005*	*-0.022, 0.029*	*0.69*	
Nutrition knowledge	Lower wealth	0.075	0.016, 0.138		17.3%
	Higher wealth	0.063	0.008, 0.114		15.0%
	Difference (H−L)	*-0.012*	*-0.096, 0.066*	*0.76*	
Nutrition knowledge w/market	Lower wealth	0.085	0.026, 0.149		19.4%
	Higher wealth	0.067	0.013, 0.119		16.0%
	Difference (H−L)	*-0.017*	*-0.101, 0.061*	*0.67*	
Market activity	Lower wealth	0.026	0.005, 0.056		6.0%
	Higher wealth	0.032	0.011, 0.059		7.6%
	Difference (H−L)	*0.006*	*-0.031, 0.040*	*0.75*	

Note: Estimates are unstandardized effects with 95% Monte Carlo confidence intervals. The structural equation model controlled for the following baseline covariates as potential mediator-outcome confounders: religion, log-transformed homestead land size, log-transformed agricultural land size, garden crop species richness, market score, women's education, domains of women's empowerment (including mobility, social support, communication with husband, external communication, own income, and decision-making), and women's dietary diversity score. We estimated the model using Full Information Maximum Likelihood (FIML) and allowed for correlations between the residuals of all the poultry and garden mediators and the residuals of the knowledge mediators. The model includes 2612 women. Fit statistics: CFI=0.993; TLI=0.856; RMSEA=0.044; $\chi^2(16)=57.0$, p<0.001. Abbreviations: CFI – Comparative Fit Index, CI – confidence interval, RMSEA – Root Mean Square Error of Approximation, TLI – Tucker-Lewis Index, w/ - with

Supplementary Information 9. Heterogeneity in direct and indirect effects of the FAARM intervention on women's dietary diversity score, by women's education level (lower = no or partial/complete primary; higher = partial or complete secondary education).

	Group	β	95% CI	Interaction p-value	Proportion mediated
Total effect	Lower education	0.501	0.300, 0.700		
	Higher education	0.308	0.110, 0.504		
	Difference (H–L)	*-0.192*	*-0.476, 0.088*	*0.18*	
Direct effect	Lower education	-0.022	-0.195, 0.152		
	Higher education	-0.009	-0.210, 0.191		
	Difference (H–L)	*0.013*	*-0.253, 0.279*	*0.92*	
Indirect effect	Lower education	0.523	0.411, 0.640		104.4%
	Higher education	0.317	0.198, 0.445		102.9%
	Difference (H–L)	*-0.206*	*-0.377, -0.034*	*0.02*	
<u>Indirect effects by domain:</u>					
Home garden production	Lower education	0.396	0.295, 0.507		79.0%
	Higher education	0.224	0.118, 0.346		72.8%
	Difference (H–L)	*-0.171*	*-0.326, -0.013*	*0.03*	
Home garden production w/market	Lower education	0.415	0.314, 0.526		82.9%
	Higher education	0.230	0.121, 0.353		74.5%
	Difference (H–L)	*-0.186*	*-0.340, -0.026*	*0.02*	
Poultry production	Lower education	0.016	0.001, 0.035		3.1%
	Higher education	0.016	-0.004, 0.037		5.3%
	Difference (H–L)	*0.001*	*-0.027, 0.026*	*0.96*	
Poultry production w/market	Lower education	0.017	0.002, 0.036		3.4%
	Higher education	0.017	-0.003, 0.038		5.6%
	Difference (H–L)	*0.000*	*-0.027, 0.026*	*0.99*	
Nutrition knowledge	Lower education	0.072	0.018, 0.130		14.5%
	Higher education	0.060	0.011, 0.107		19.6%
	Difference (H–L)	*-0.012*	*-0.088, 0.060*	*0.74*	
Nutrition knowledge w/market	Lower education	0.086	0.030, 0.145		17.1%
	Higher education	0.062	0.013, 0.109		20.1%
	Difference (H–L)	*-0.024*	*-0.100, 0.049*	*0.52*	
Market activity	Lower education	0.039	0.015, 0.067		7.8%
	Higher education	0.016	0.000, 0.040		5.2%
	Difference (H–L)	*-0.023*	*-0.056, 0.010*	*0.16*	

Note: Estimates are unstandardized effects with 95% Monte Carlo confidence intervals. The structural equation model controlled for the following baseline covariates as potential mediator-outcome confounders: religion, baseline wealth quintile, homestead land size, agricultural land size, garden crop species richness, market score, domains of women's empowerment (including mobility, social support, communication with husband, external communication, own income, and decision-making), and women's dietary diversity score. We estimated the model using Full Information Maximum Likelihood (FIML) and allowed for correlations between the residuals of all the poultry and garden mediators and the residuals of the knowledge mediators. The model includes 2612 women. Fit statistics: CFI=0.993; TLI=0.858; RMSEA=0.044; $\chi^2(16)=57.2$, $p<0.001$. Abbreviations: CFI – Comparative Fit Index, CI – confidence interval, RMSEA – Root Mean Square Error of Approximation, TLI – Tucker-Lewis Index, w/ - with

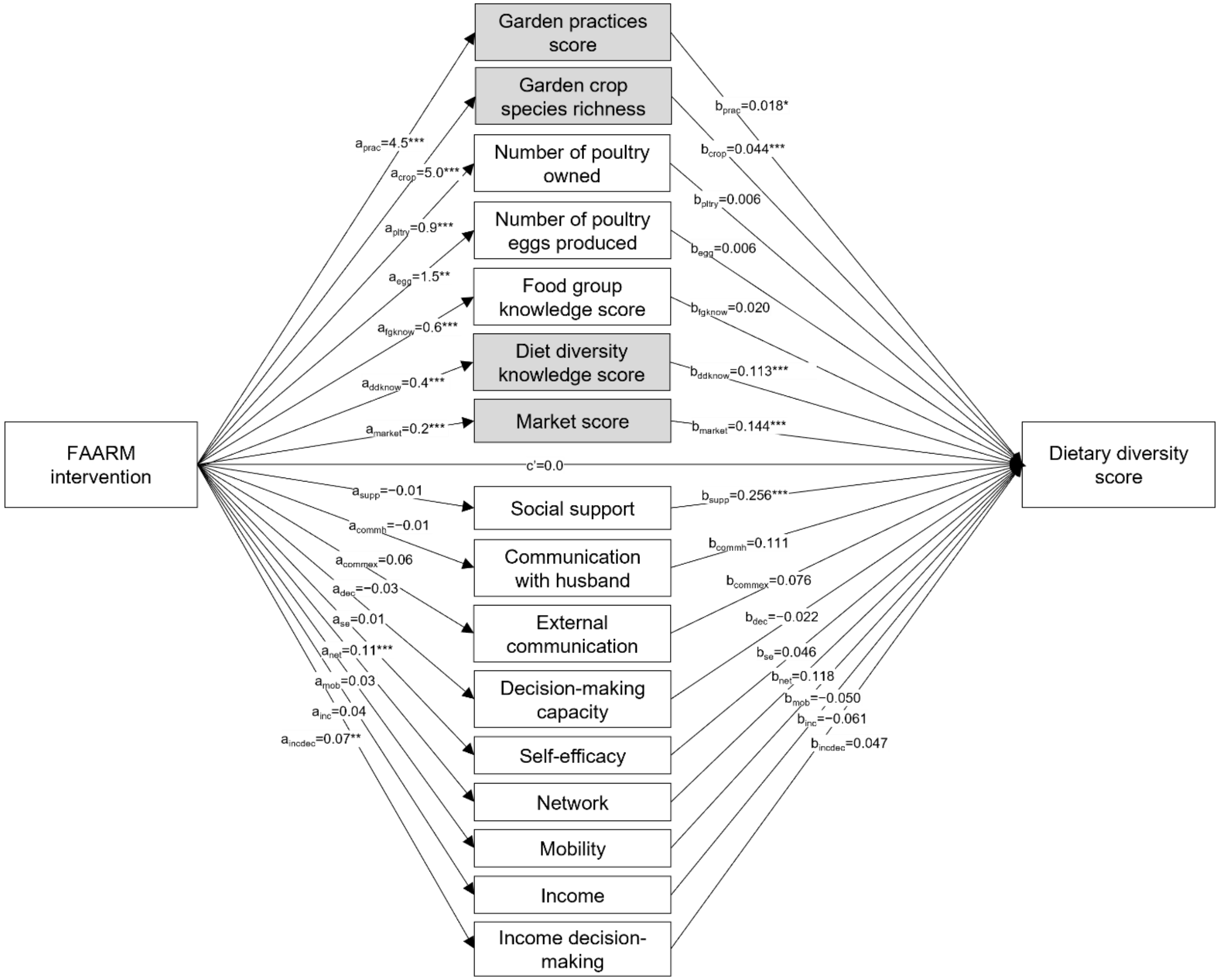

Supplementary Information 10. Causal mediation pathways of the FAARM intervention on women's dietary diversity score through women's home garden production, poultry production, nutrition knowledge, market activity, and empowerment.
Path coefficients estimated using structural equation modeling (n=2,612) with Full Information Maximum Likelihood and cluster-adjusted standard errors, controlling for baseline covariates. Unstandardized coefficients of the *a* paths are the effect of the intervention on the mediators and coefficients of the *b* paths are the effect of a one-unit increase in the mediators on dietary diversity score. Residual correlations and variances are omitted from the figure. Corresponding indirect effects are included in Supplementary Information 11. P-values are estimated using two-sided Wald tests. P-values: *p<0.05, **p<0.01, ***p<0.001; mediators are shaded grey if the indirect effect through the mediator has a p-value below 0.05.

Supplementary Information 11. Direct and indirect effects of the FAARM intervention on women's dietary diversity score through women's home garden production, poultry production, nutrition knowledge, market activity, and empowerment (as shown in Supplementary Information 10).

	**β**	**95% CI**	**Proportion mediated**
Total effect[a]	0.423	0.239, 0.599	
Direct effect	0.000	-0.162, 0.161	
Indirect effect[b]	0.422	0.316, 0.528	99.9%
Individual indirect effects:			
Home garden production			
Garden practices score (a_{prac}*b_{prac})	0.082	0.014, 0.154	
Garden crop species richness (a_{crop}*b_{crop})	0.220	0.155, 0.296	
Poultry production			
No. of poultry owned (a_{pltry}*b_{pltry})	0.005	-0.009, 0.019	
No. of poultry eggs produced (a_{egg}*b_{egg})	0.010	0.000, 0.022	
Nutrition knowledge			
Food group knowledge score (a_{fgknow}*b_{fgknow})	0.012	-0.017, 0.040	
Diet diversity knowledge score (a_{ddknow}*b_{ddknow})	0.045	0.019, 0.078	
Market activity			
Market score (a_{market}*b_{market})	0.033	0.015, 0.057	
Empowerment			
Social support score (a_{supp}*b_{supp})	-0.003	-0.031, 0.020	
Communication with husband score (a_{commh}*b_{commh})	-0.001	-0.009, 0.003	
External communication score (a_{commex}*b_{commex})	0.004	-0.006, 0.018	
Decision-making score (a_{dec}*b_{dec})	0.001	-0.001, 0.005	
Self-efficacy score (a_{se}*b_{se})	0.000	-0.003, 0.004	
Network score (a_{net}*b_{net})	0.013	-0.002, 0.031	
Mobility (a_{mob}*b_{mob})	-0.001	-0.007, 0.002	
Own income (a_{inc}*b_{inc})	-0.002	-0.010, 0.002	
Decision-making on income (a_{incdec}*b_{incdec})	0.003	-0.002, 0.011	
Indirect effects by domain:			
Home garden production (∑prac + crop)	0.303	0.222, 0.390	71.6%
Poultry production (∑pltry + egg)	0.015	0.002, 0.030	3.5%
Nutrition knowledge (∑fgknow + ddknow)	0.057	0.017, 0.099	13.6%
Market activity (market score)	0.033	0.015, 0.057	7.9%
Empowerment (∑supp + commh + commex + dec + se + net + mob + inc + incdec)	0.014	-0.029, 0.051	3.4%

Note: Estimates are unstandardized effects with 95% Monte Carlo confidence intervals. The structural equation model controlled for the following baseline covariates as potential mediator-outcome confounders: religion, household wealth quintile, log-transformed homestead land size, log-transformed agricultural land size, garden crop species richness, market score, women's education, domains of women's empowerment (including mobility, social support, communication with husband, external communication, own income, and decision-making), and women's dietary diversity score. We estimated the model using Full Information Maximum Likelihood (FIML). We allowed for correlations between the residuals of all the poultry and garden mediators, the residuals of the knowledge mediators, and the residuals of all the empowerment mediators and the market mediator. The model includes 2612 women.

Fit statistics: CFI=0.947; TLI=0.614; RMSEA=0.051; $\chi^2(68)=534.8$, $p<0.001$

[a]The total effect is the sum of the direct effect and the total indirect effect.

[b]The indirect effect is calculated as the sum of the indirect effects through garden production, poultry production, nutrition knowledge, market activity, and empowerment.

Abbreviations: CFI – Comparative Fit Index, CI – confidence interval, No. - number, RMSEA – Root Mean Square Error of Approximation, TLI – Tucker-Lewis Index

Supplementary Information 12. Direct and indirect effects of the FAARM intervention on women's dietary diversity score, excluding women with observations during Ramadan (sensitivity analysis).

	**β**	**95% CI**	**Proportion mediated**
Total effect[a]	0.417	0.239, 0.589	
Direct effect	0.002	-0.156, 0.160	
Indirect effect[b]	0.414	0.320, 0.514	99.4%
<u>*Individual indirect effects:*</u>			
Home garden production			
Garden practices score (a_{prac}*b_{prac})	0.073	0.003, 0.148	
Garden crop species richness (a_{crop}*b_{crop})	0.227	0.161, 0.301	
Garden practices via market (a_{prac}*d_{prac}*b_{market})	-0.010	-0.020, -0.001	
Garden crops via market (a_{crop}*d_{crop}*b_{market})	0.022	0.011, 0.037	
Poultry production			
No. of poultry owned (a_{pltry}*b_{pltry})	0.007	-0.006, 0.021	
No. of poultry eggs produced (a_{egg}*b_{egg})	0.008	-0.002, 0.020	
Poultry owned via market (a_{pltry}*d_{pltry}*b_{market})	0.002	0.000, 0.005	
Poultry eggs via market (a_{egg}*d_{egg}*b_{market})	-0.001	-0.003, 0.001	
Nutrition knowledge			
Food group knowledge score (a_{fgknow}*b_{fgknow})	0.018	-0.011, 0.044	
Diet diversity knowledge score (a_{ddknow}*b_{ddknow})	0.051	0.023, 0.086	
Food group score via market (a_{fgknow}*d_{fgknow}*b_{market})	0.006	0.001, 0.011	
Diet diversity score via market (a_{ddknow}*d_{ddknow}*b_{market})	0.001	-0.001, 0.004	
Market activity			
Market score (a_{market}*b_{market})	0.010	-0.006, 0.028	
Production/knowledge via market (∑a*d*b paths)	0.021	0.010, 0.036	
<u>*Indirect effects by domain:*</u>			
Home garden production (∑prac+crop)	0.299	0.219, 0.390	71.9%
Home garden production w/market (∑prac+crop+garden prod via market)	0.312	0.229, 0.406	74.9%
Poultry production (∑pltry+egg)	0.015	0.003, 0.028	3.6%
Poultry production w/market (∑pltry+egg+poultry prod via market)	0.017	0.004, 0.031	4.0%
Nutrition knowledge (∑fgknow + ddknow)	0.069	0.028, 0.112	16.6%
Nutrition knowledge w/market (∑know+know via market)	0.076	0.035, 0.120	18.2%
Market activity (∑market+prod/know via market)	0.031	0.013, 0.054	7.4%

Note: The structural equation model controlled for the following baseline covariates as potential mediator-outcome confounders: religion, household wealth quintile, log-transformed homestead land size, log-transformed agricultural land size, garden crop species richness, women's education, domains of women's empowerment (including mobility, social support, communication with husband, external communication, own income, and decision-making, social support, communication, and decision-making), and women's dietary diversity score. We estimated the model using Full Information Maximum Likelihood (FIML). We allowed for correlations between the residuals of all the poultry and garden mediators, the residuals of the knowledge mediators, and the residuals of the empowerment mediators. The model includes 2611 women.
Fit statistics: CFI=0.993; TLI=0.834; RMSEA=0.045; $\chi^2(8)=49.4$, $p<0.001$
[a]The total effect is the sum of the direct effect and the total indirect effect.
[b]The indirect effect is calculated as the sum of the indirect effects through garden production, poultry production, nutrition knowledge, and market activity. Abbreviations: CFI – Comparative Fit Index, CI – confidence interval, No. - number, RMSEA – Root Mean Square Error of Approximation, TLI – Tucker-Lewis Index, w/ - with

Supplementary Information 13. Supplementary methods.

Intervention description

To facilitate program activities, women were organized into village-level 'women farmer groups' ranging in size from 8 to 26 women. In each group, one woman and her family were elected as lead farmers. Over three years, groups were trained on different project components with sessions held about every two months (see below). Women also received individually tailored counseling visits at home from field staff approximately every other month. When the project closed, each group selected one or more peer educators to continue to support promoted activities.

The project activities are listed below by intervention component.

Home gardening: With a goal of year-round vegetable production, women received seasonally focused training on homestead vegetable and fruit production as well as sustainable gardening techniques such as fertilizer production, seed preservation and selection, and sack gardening. In addition to maintaining a model farm and a space for training, lead farmers nurtured seedlings and saplings for distribution to other participants. Seeds for nutrient-rich, local vegetables were provided to households once or twice per year during the main planting seasons, and interested households with sufficient land also received fruit tree saplings. Participants also received small gardening assets such as watering cans and spades.

Poultry rearing: Poultry training was provided annually and included information on how to raise healthy poultry and chicks, meet their nutritional needs, manage common diseases through deworming and vaccination, and construct an 'improved' poultry shed. Participants received starter feed and watering stations, and partial reimbursement to build the shed and to purchase 3-4 poultry from local vendors. A community vaccination program was established to provide participating households with the opportunity to vaccinate their poultry against common poultry diseases at low cost.

Market links: Market training was provided at the start of the third year of the intervention with the goal of enabling participating women to sell surplus garden and poultry products through collection points using a group marketing approach. Marketing sessions held subsequent to the training were organized by the lead farmer families in each area.

Nutrition counseling: Women and their families received courtyard training sessions every two months covering topics in women's nutrition, prevention of anemia, vitamin A deficiency, iodine deficiency, infant and young child feeding practices, sick child care, and hygiene. An additional food hygiene component was incorporated from June 2017 to February 2018 which emphasized hand-washing, clean utensils, food storage, and food reheating.[1]

[1]Sobhan, Shafinaz, Anna A. Müller-Hauser, Tarique Md. Nurul Huda, Jillian L. Waid, Om Prasad Gautam, Giorgia Gon, Amanda S. Wendt, and Sabine Gabrysch. "Design, Delivery, and Determinants of Uptake: Findings from a Food Hygiene Behavior Change Intervention in Rural Bangladesh." *BMC Public Health* 22, no. 1 (May 4, 2022): 887. https://doi.org/10.1186/s12889-022-13124-w.

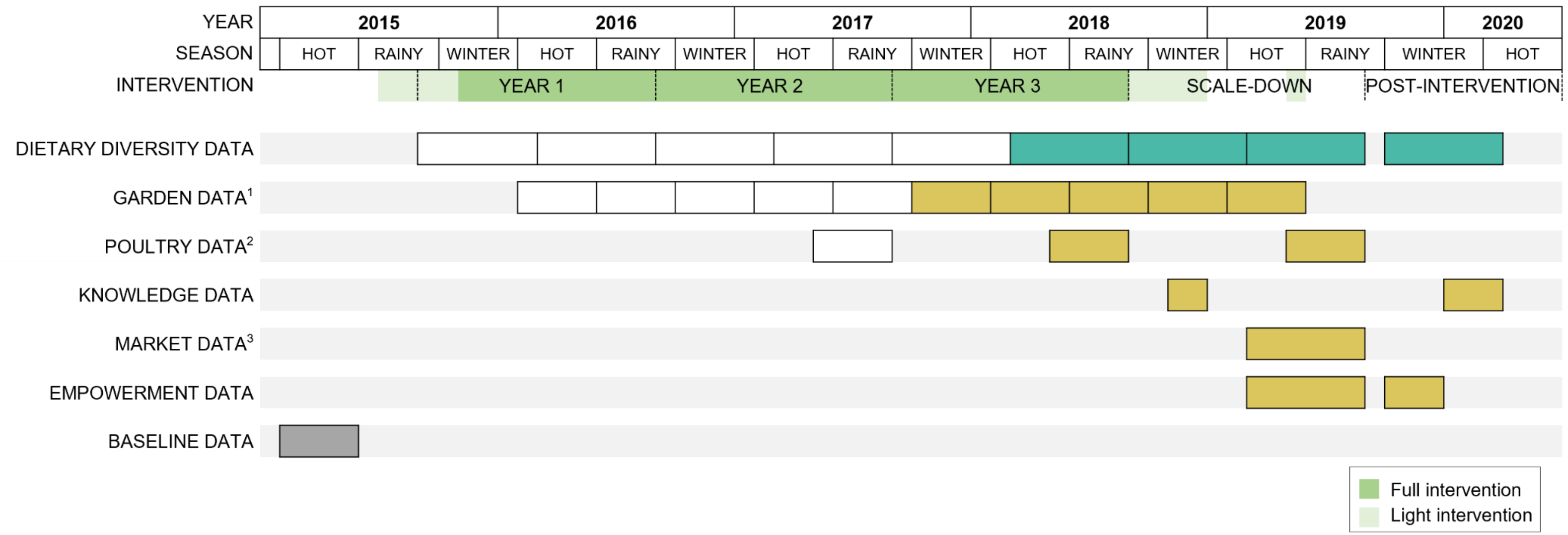

Supplementary Information 14. Timeline of intervention activities and data collection periods.
Shaded boxes represent the data collection periods of the data used in this mediation analysis and the unshaded boxes all available data periods as part of the FAARM trial surveillance system and endline survey. The outcome is shaded in green, the mediators in yellow, and the confounders in grey. The darker green shading for the intervention (full intervention) indicates greater intervention programming intensity during the depicted time periods. [1]For garden data, boxes represent the recall period and not the time of the interview (i.e., garden questions captured information on the prior season). [2]Poultry questions captured information on the prior week. [3]Market questions captured information on the prior month.

Supplementary Information 15. Variables included in the mediation analysis.

Variable	Function	Level	Data collection	Analytic sample size	Description
**FAARM intervention**	Exposure	Cluster	N/A	2612	Random allocation to the intervention or control group
**Dietary diversity score**	Outcome	Individual	Routine assessment, Mar 2018-Feb 2020 (4 rounds)	2612	The number of food groups consumed (out of 10) by a woman in the prior 24 hours, averaged over four data collection rounds (or the number of rounds completed if missing data).[2] Score 0-10.
**Minimum dietary diversity**	Outcome	Individual	Routine assessment, Mar 2018-Feb 2020 (4 rounds)	2612	The proportion of times a woman met the criteria for minimum dietary diversity (≥5 out of 10 food groups in the prior 24 hours) over four data collection rounds (or the number of rounds completed if missing data).[1] Score 0.0-1.0.
**Garden crop species richness**	Mediator	Individual	Routine assessment, Mar 2018-Sep 2019 (5 rounds)	2465	The number of crop species harvested in a woman's home garden in the prior season (4 months), averaged over five data collection rounds (or the number of rounds completed if missing data).
**Garden practices score**	Mediator	Individual	Routine assessment, Mar-June 2018 (1 round)	2389	The number of promoted garden practices applied (out of 17). Garden practices included raised beds, intercropping, seed beds, seed storage, integrated pest management, organic fertilizer, fencing, live fencing, pit crops, drought-resistant plants, flood-resistant plants, windbreak/protective trees, fruit trees,

[2] FAO and FHI 360. Minimum Dietary Diversity for Women: A Guide to Measurement. Rome: FAO, 2016

					sapling production, seedling production, biochar-based fertilizer, and sack gardening. Score 0-17.
**Number of poultry owned**	Mediator	Individual	Routine assessment, May-Sep 2018/2019 (2 rounds)	2447	The number of poultry owned at the time of the interview, averaged over two data collection rounds. The number of poultry was capped at 95 for each round to reduce the influence of outliers[3].
**Number of poultry eggs produced**	Mediator	Individual	Routine assessment, May-Sep 2018/2019 (2 rounds)	2447	The number of poultry eggs produced in the past week averaged over two data collection rounds. The number of eggs was capped at 95 for each round to reduce the influence of outliers[3].
**Dietary diversity knowledge score**	Mediator	Individual	Routine assessment Nov-Dec 2018 (1 round)	2363	Sum score for naming types of foods (0-10) an adult woman should eat regularly (scored as 0 for 0-3, 1 for 4-6, 2 for 7-10)[4] and reasons why a diverse diet is especially important for a woman during pregnancy and lactation (scored as 0 for 0, 1 for 1-2, 2 for 2-5)[5]. Score 0-4.
**Food group knowledge score**	Mediator	Individual	Endline (ended early due to COVID-19), Jan-Mar 2020 (1 round)	1765	The number of correct answers to five multiple-choice nutrition questions, asking the correct health reason (giving energy, building the body and giving strength, or protecting from disease) for eating dark green leafy vegetables, animal foods, yellow and orange fruits/vegetables, pulses, and staples. Score 0-5.

[3] Lambrecht, N. J. et al. Impact of a Homestead Food Production program on poultry rearing and egg consumption: A cluster-randomized controlled trial in Bangladesh. Maternal & Child Nutrition e13505 (2023) doi:10.1111/mcn.13505.
[4] Leafy vegetables (shak), fleshy vegetables (shobji), vegetables (shakshobji), milk or dairy products, a piece of meat/fish, egg, pulses (dal), a colored/seasonal fruit, rice/cereal, other (for other food type).
[5] To ensure that the baby grows well, to give birth to a healthy baby, to provide nutritious breast milk to the growing baby, to be healthy and energetic, other reason.

**Market score**	Mediator	Individual	Routine assessment, Mar-Aug 2019 (1 round)	2337	A woman’s market activity based on the sum score of a woman buying goods (yes/no), going to the market to buy goods (yes/no), and selling goods (yes/no) in the prior month. Score 0-3.
**Mobility**	Mediator (Supplemental analysis)	Individual	Routine assessment, Mar-Aug 2019 (1 round)	2337	Whether a woman had left the homestead in the prior month (yes/no).
**Social support score**	Mediator (Supplemental analysis)	Individual	Routine assessment, Mar-Aug 2019 (1 round)	2337	Sum score of a woman having somebody who can help when in need through: lending her money, giving her food, visiting/helping when sick, talking over problems, and whether she had frequent contact with her natal family. Score scaled to 0-2 (from 0-5).
**Communication with husband score**	Mediator (Supplemental analysis)	Individual	Routine assessment, Mar-Aug 2019 (1 round)	2313	Sum score of topics a woman discusses often or sometimes with her husband: work/agricultural activities, events at home, expenditures, community events, and her own health. Score scaled to 0-2 (from 0-5).
**External communication score**	Mediator (Supplemental analysis)	Individual	Routine assessment, Mar-Aug 2019 (1 round)	2337	Sum score of topics a woman usually discusses with other women: problems of the community, education problems, health problems, women's issues, and information on health/nutrition, and whether she is at least fairly comfortable speaking up in a meeting where men and women are present. Score scaled to 0-2 (from 0-6).
**Decision-making score**	Mediator (Supplemental analysis)	Individual	Endline, Oct-Dec 2019 (1 round)	2513	Sum score of a woman’s usual involvement in decision-making on food preparation, household purchases for daily needs, major household purchases, her visits to her family or relatives, and her

					own health care. Score scaled to 0-2 (from 0-5).
**Self-efficacy score**	Mediator (Supplemental analysis)	Individual	Endline, Oct-Dec 2019 (1 round)	2556	A woman's "perception of her capabilities and ability to reach her goals" based on the sum score of 8 statements[4], each scored 1-5: strongly disagree (1), disagree (2), neither agree or disagree (3), agree (4), or strongly agree (5). Score (8-40) dichotomized, 1 if >=32, 0 if <32[5].
**Network score**	Mediator (Supplemental analysis)	Individual	Endline, Oct-Dec 2019 (1 round)	2560	Sum of the number of relationships women had, topics discussed, and support available from a random selection of 5 women in her settlement. Score scaled to 0-2 with 2 being the highest score in the sample (from 0 to 53).
**Own income**	Mediator (Supplemental analysis)	Individual	Routine assessment, Mar-Aug 2019 (1 round)	2337	Whether a woman earned any money in the prior month (yes/no).
**Income decision-making**	Mediator (Supplemental analysis)	Individual	Endline, Oct-Dec 2019 (1 round)	2513	Whether a woman decides on her own earnings (yes/no). Coded 0 if the woman was not a decision-maker on her earnings or had no earnings.
**Dietary diversity score (baseline)**	Confounder	Individual	Baseline, Mar-May 2015	2612	The number of food groups consumed (out of 10) by a woman in the prior 24 hours, at baseline. Women with missing data (n=101) were assigned the mean dietary

[4] Statements: 1) I will be able to achieve most of the goals that I have set for myself, 2) When facing difficult tasks, I am certain that I will accomplish them, 3) In general, I think that I can obtain outcomes that are important to me, 4) I believe I can succeed at most any endeavor to which I set my mind, 5) I will be able to successfully overcome many challenges, 6) I am confident that I can perform effectively on many different tasks, 7) Compared to other people, I can do most tasks very well, 8) Even when things are tough, I can perform quite well. Reference: Chen, G., Gully, S. M., & Eden, D. (2001). Validation of a new general self-efficacy scale. *Organizational Research Methods, 4*(1), 62–83. https://doi.org/10.1177/109442810141004

[5] Chen, G., Gully, S. M., & Eden, D. (2001). Validation of a new general self-efficacy scale. *Organizational Research Methods, 4*(1), 62–83. https://doi.org/10.1177/109442810141004

					diversity score of their settlement. Score 0-10.
**Household wealth quintile**	Confounder	Household	Baseline, Mar-May 2015	2612	Quintiles of household wealth, based on a wealth index calculated using principal components analysis of household assets. Women with missing data (n=11) were assigned the wealth quintile of their settlement.
**Household religion**	Confounder	Household	Baseline, Mar-May 2015	2612	Muslim or Hindu
**Homestead land size**	Confounder	Household	Baseline, Mar-May 2015	2612	Size of homestead land (in decimals[6]). Women with missing data (n=12) were assigned the mean value of their settlement.
**Agricultural land size**	Confounder	Household	Baseline, Mar-May 2015	2612	Size of agricultural land (in decimals). Women with missing data (n=12) were assigned the mean value of their settlement.
**Garden crop species richness**	Confounder	Household	Baseline, Mar-May 2015	2612	The number of crop species harvested in a woman's home garden in the prior year of 53 possible harvested garden crop species. Women with missing data (n=12) were assigned the mean value of their settlement.
**Market score**	Confounder	Individual	Baseline, Mar-May 2015	2612	A woman’s market activity based on the sum score of a woman buying goods (yes/no), going to the market to buy goods (yes/no), and selling goods (yes/no) in the prior month. Score 0-3. Women with missing data (n=16) were assigned the mean score of their settlement.

[6] A decimal is land area unit equal to approximately 40.47 square meters.

**Education level**	Confounder	Individual	Baseline, Mar-May 2015	2612	A woman’s education level based on her reported number of completed school years: no formal education (0), partial primary (1), complete primary (2), partial secondary (3), or complete secondary education and higher (4).
**Mobility**	Confounder	Individual	Baseline, Mar-May 2015	2612	Whether a woman had left the homestead at least once in the prior month (yes/no). Women with missing data (n=21) were assigned the mean score of their settlement.
**Social support score**	Confounder	Individual	Baseline, Mar-May 2015	2612	Sum score of a woman having somebody who can help when in need through: lending her money, giving her food, visiting/helping when sick, talking over problems, and whether she had frequent contact with her natal family. Women with missing data (n=21) were assigned the mean score of their settlement. Score scaled to 0-2 (from 0-5).
**Communication with husband score**	Confounder	Individual	Baseline, Mar-May 2015	2612	Sum score of topics a woman discusses often or sometimes with her husband: work/agricultural activities, events at home, expenditures, community events, and her own health. Women with missing data (n=34) were assigned the mean score of their settlement. Score scaled to 0-2 (from 0-5).
**External communication score**	Confounder	Individual	Baseline, Mar-May 2015	2612	Sum score of topics a woman usually discusses with other women: problems of the community, education problems, health problems, women's issues, and information on health/nutrition, and whether she is at least fairly comfortable speaking up in a meeting where men and women are present. Women with missing data (n=21)

					were assigned the mean score of their settlement. Score scaled to 0-2 (from 0-6).
**Own income**	Confounder	Individual	Baseline, Mar-May 2015	2612	Whether a woman earned any money in the prior month (yes/no). Women with missing data (n=21) were assigned the mean score of their settlement.
**Decision-making score**	Confounder	Individual	Baseline, Mar-May 2015	2612	Sum score of a woman’s usual involvement in decision-making on food preparation, household purchases for daily needs, major household purchases, her visits to her family or relatives, and her own health care. Women with missing data (n=21) were assigned the mean score of their settlement. Score scaled to 0-2 (from 0-5).

Note: For both the confounder and mediator variables social support score, communication with husband score, external communication score, decision-making score, and network score, scores are re-scaled linearly from 0-2 with 0 being the lowest score in the sample and 2 being the highest score.
