# Supplementary material for: Impact pathways of a homestead food production programme on women’s dietary diversity in Bangladesh
Source: Nat Food. 2026 May 12;7(5):464–73. doi: 10.1038/s43016-026-01354-9 (PMC13212147; doi:10.1038/s43016-026-01354-9)
Supplement: Supplementary file 2 — Reporting Summary [file 43016_2026_1354_MOESM2_ESM.pdf]

nature portfolio

Corresponding author(s): Nathalie Lambrecht

Last updated by author(s): Apr 9, 2026

Reporting Summary

Nature Portfolio wishes to improve the reproducibility of the work that we publish. This form provides structure for consistency and transparency in reporting. For further information on Nature Portfolio policies, see our Editorial Policies and the Editorial Policy Checklist.

Statistics

For all statistical analyses, confirm that the following items are present in the figure legend, table legend, main text, or Methods section.

n/a	Confirmed	
☐	☒	The exact sample size (*n*) for each experimental group/condition, given as a discrete number and unit of measurement
☐	☒	A statement on whether measurements were taken from distinct samples or whether the same sample was measured repeatedly
☐	☒	The statistical test(s) used AND whether they are one- or two-sided<br>*Only common tests should be described solely by name; describe more complex techniques in the Methods section.*
☐	☒	A description of all covariates tested
☐	☒	A description of any assumptions or corrections, such as tests of normality and adjustment for multiple comparisons
☐	☒	A full description of the statistical parameters including central tendency (e.g. means) or other basic estimates (e.g. regression coefficient) AND variation (e.g. standard deviation) or associated estimates of uncertainty (e.g. confidence intervals)
☐	☒	For null hypothesis testing, the test statistic (e.g. *F*, *t*, *r*) with confidence intervals, effect sizes, degrees of freedom and *P* value noted<br>*Give P values as exact values whenever suitable.*
☒	☐	For Bayesian analysis, information on the choice of priors and Markov chain Monte Carlo settings
☐	☒	For hierarchical and complex designs, identification of the appropriate level for tests and full reporting of outcomes
☒	☐	Estimates of effect sizes (e.g. Cohen's *d*, Pearson's *r*), indicating how they were calculated

Our web collection on statistics for biologists contains articles on many of the points above.

Software and code

Policy information about availability of computer code

Data collection	We collected data on 2705 women as part of the Food and Agricultural Approaches to Malnutrition (FAARM) trial in Sylhet division, Bangladesh from 2015 to 2020. Data were collected on tablets using the open data kit platform. https://doi.org/10.1145/2369220.2369236
Data analysis	Data processing and analyses were conducted using Stata MP version 18.0 (StataCorp, College Station, TX) and structural equation models were fit in R (version 4.5.0) using the 'lavaan' package (0.6-10). https://doi.org/10.18637/jss.v048.i02 Monte Carlo 95% confidence intervals were calculated using semTools(0.5-7). https://CRAN.R-project.org/package=semTools

For manuscripts utilizing custom algorithms or software that are central to the research but not yet described in published literature, software must be made available to editors and reviewers. We strongly encourage code deposition in a community repository (e.g. GitHub). See the Nature Portfolio guidelines for submitting code & software for further information.

Data

Policy information about availability of data

All manuscripts must include a data availability statement. This statement should provide the following information, where applicable:

- Accession codes, unique identifiers, or web links for publicly available datasets
- A description of any restrictions on data availability
- For clinical datasets or third party data, please ensure that the statement adheres to our policy

The aggregated dataset for the replication of this study will be made available online. A deidentified dataset with the individual participant response data that

underlie the results reported in this article is available upon request. Interested researchers will need to provide a methodologically sound proposal for use of the panel data and sign a data access agreement to gain access to the data. The underlying individual response data are not publicly available due to privacy restrictions and their complexity. Data requests with a proposal should be directed to the corresponding author (NJL) and the principal investigator (SG; sabine.gabrysch@charite.de). The FAARM trial protocol is available online.

Research involving human participants, their data, or biological material

Policy information about studies with human participants or human data. See also policy information about sex, gender (identity/presentation), and sexual orientation and race, ethnicity and racism.

Reporting on sex and gender	We report only on outcomes pertaining to women as the FAARM trial was designed to target the nutrition and health of women (and their children). The intervention was implemented using woman farmer groups and individual counseling sessions for women.
Reporting on race, ethnicity, or other socially relevant groupings	We report on participant's religion (self-report) and household wealth quintile (calculated using principle components analysis of household assets). We control for the following baseline covariates as potential mediator-outcome confounders in the SEM analysis selected a priori based on theory: women's DDS at baseline, household wealth quintile at baseline (based on household assets), household religion (Muslim or Hindu), homestead and agricultural land size, garden crop species richness at baseline, market score at baseline, women's education level (completed school years), and indicators of women's empowerment at baseline.
Population characteristics	We describe population characteristics in Table 1. Women included in our analysis were, on average, 25 years old. Two-thirds were Muslim and one-third Hindu. About 15% had no education, approximately half had partial or complete primary education, one-third had partial secondary education, and about 6% had complete secondary education.
Recruitment	All households in the study sites of Baniachong and Nabiganj subdistricts were enumerated to identify women eligible to participate in the FAARM trial. Eligibility criteria included: a self-reported age of 30 years or younger, married, access to at least 40 square-meters of land, and interest in participating in a Homestead Food Production program. Geographical clusters were formed based on the location of women's residences, resulting in 96 settlements comprising 2895 eligible women. Eligible women in the clusters were then approached for consent. Women who had migrated since enumeration (n=91), were repeatedly not at home (n=9), were deceased (n=3), had divorced/separated/widowed (n=46), had a disability (n=3), or refused (n=119) were excluded. One woman was mistakenly excluded. 2623 women were enrolled. After baseline, the 96 settlements were randomly allocated 1:1 to intervention and control. In the second year of the trial, an additional 82 women who had newly married into participant households were enrolled: 35 in the intervention and 47 in the control arm.<br>At endline, over 4 years later, 2579 of 2705 enrolled women could be reached (94% in control and 96% in intervention). Our analytic sample consists of observations from 2612 women (1312 control, 1300 intervention), reached during the trial and/or at endline. As 97% of enrolled women provided data for this analysis, there is limited scope for selection bias.<br>(See Appendix 1 for the flowchart)
Ethics oversight	The FAARM trial protocol was positively reviewed by ethics committees at Heidelberg University's Medical Faculty in Germany (Reference: S-121/2014) and the James P. Grant School of Public Health, BRAC University in Bangladesh (Ref.: 37A). Written informed consent was obtained from all study participants before data collection.

Note that full information on the approval of the study protocol must also be provided in the manuscript.

Field-specific reporting

Please select the one below that is the best fit for your research. If you are not sure, read the appropriate sections before making your selection.

☐ Life sciences ☒ Behavioural & social sciences ☐ Ecological, evolutionary & environmental sciences

For a reference copy of the document with all sections, see nature.com/documents/nr-reporting-summary-flat.pdf

Behavioural & social sciences study design

All studies must disclose on these points even when the disclosure is negative.

Study description	The study was a 1:1 cluster-randomized controlled trial. We use quantitative, panel data from the trial and structural equation modeling methods to estimate the impact pathways of the Homestead Food Production intervention on women's dietary diversity.
Research sample	The sample includes married women who reported to be less than 30 years old at the time of enrollment, living in Nabiganj and Baniachong subdistricts, Habiganj District, Sylhet Division, Bangladesh. This study area was chosen because it has a high prevalence of food insecurity and undernutrition, as well as suitability for gardening. We included married women of the selected age group to ensure that a sufficient number of infants would be born to the women during the trial period to have enough statistical power to detect effects on the primary outcome of the trial, child length-for-age. Women included in our analysis were, on average, 25 years old. Two-thirds were Muslim and one-third Hindu. About 15% had no education, approximately half had partial or complete primary education, one-third had partial secondary education, and about 6% had complete secondary education. The sample is not representative of women living in Sylhet Dvision.
Sampling strategy	The study sample size was calculated based on the trial's primary outcome, child length-for-age z-score, with a target enrollment of approximately 1490 children. This sample size allows for a minimal detectable effect size of a 0.3 food groups difference in dietary

diversity score for women. No additional sample size calculations were conducted for the mediation analysis. All households in the study sites of Baniachong and Nabiganj subdistricts were enumerated to identify women eligible to participate in the FAARM trial. Eligibility criteria included: a self-reported age of 30 years or younger, married, access to at least 40 square-meters of land, and interest in participating in a Homestead Food Production program. Geographical clusters were formed based on the location of women's residences, resulting in 96 settlements comprising 2895 eligible women. Eligible women in the clusters were then approached for consent. Women who had migrated since enumeration (n=91), were repeatedly not at home (n=9), were deceased (n=3), had divorced/separated/widowed (n=46), had a disability (n=3), or refused (n=119) were excluded. One woman was mistakenly excluded. 2623 women were enrolled. After baseline, the 96 settlements were randomly allocated 1:1 to intervention and control. In the second year of the trial, an additional 82 women who had newly married into participant households were enrolled: 35 in the intervention and 47 in the control arm.

Data collection	Data were collected on tablets by trained data collection officers using face-to-face interviews. Enumerators were separate from the implementation team and were blind to the experimental allocation of the clusters. Enumerators were given a 2-week training before baseline data collection and 1- to 2-day refresher trainings before each round of data collection during the routine surveillance system data collection.
Timing	Baseline data were collected from March to May 2015, a surveillance system was implemented to periodically collect data from September 2015 to August 2019, and endline data were collected from September 2019 to April 2020.
Data exclusions	We excluded data from women if they provided no outcome data during the analyzed time period (n=93).
Non-participation	At endline, 94% of women in the control arm and 96% of women in the intervention arm were reached. Reasons for not reaching women include death (n=16), migration (n=91), divorced/widowed (n=11), refused (n=8), and other reason (n=1).
Randomization	The 96 settlements (geographic clusters separated by a 400m buffer) were randomized 1:1 to the control and intervention group using covariate-constrained randomization.

Reporting for specific materials, systems and methods

We require information from authors about some types of materials, experimental systems and methods used in many studies. Here, indicate whether each material, system or method listed is relevant to your study. If you are not sure if a list item applies to your research, read the appropriate section before selecting a response.

Materials & experimental systems

n/a	Involved in the study	
☒	☐	Antibodies
☒	☐	Eukaryotic cell lines
☒	☐	Palaeontology and archaeology
☒	☐	Animals and other organisms
☐	☒	Clinical data
☒	☐	Dual use research of concern
☒	☐	Plants

Methods

n/a	Involved in the study	
☒	☐	ChIP-seq
☒	☐	Flow cytometry
☒	☐	MRI-based neuroimaging

Clinical data

Policy information about clinical studies

All manuscripts should comply with the ICMJE guidelines for publication of clinical research and a completed CONSORT checklist must be included with all submissions.

Clinical trial registration	ClinicalTrials.gov ID: NCT025-05711
Study protocol	https://doi.org/10.1136/bmjopen-2019-031037
Data collection	Study setting: Baniachong and Nabiganj subdistricts of Habiganj district, Sylhet division, Bangladesh<br>Period of recruitment: March to May 2015<br>Period of data collection: March 2015 to April 2020
Outcomes	The trial primary outcome was child length/height-for-age z-score. This measure was not included in this analysis. Our analytic outcome was women's dietary diversity score, a secondary outcome of the trial. Women were asked about all foods consumed in the day before the interview, and these were groups into 21 food categories by enumerators. Dietary diversity score in women was then calculated using a 10 food group score based on standard tools.

Plants

Seed stocks	*Report on the source of all seed stocks or other plant material used. If applicable, state the seed stock centre and catalogue number. If plant specimens were collected from the field, describe the collection location, date and sampling procedures.*
Novel plant genotypes	*Describe the methods by which all novel plant genotypes were produced. This includes those generated by transgenic approaches, gene editing, chemical/radiation-based mutagenesis and hybridization. For transgenic lines, describe the transformation method, the number of independent lines analyzed and the generation upon which experiments were performed. For gene-edited lines, describe the editor used, the endogenous sequence targeted for editing, the targeting guide RNA sequence (if applicable) and how the editor was applied.*
Authentication	*Describe any authentication procedures for each seed stock used or novel genotype generated. Describe any experiments used to assess the effect of a mutation and, where applicable, how potential secondary effects (e.g. second site T-DNA insertions, mosiacism, off-target gene editing) were examined.*
